# Pathological Findings in COVID-19 as a Tool to Define SARS-CoV-2 Pathogenesis. A Systematic Review

**DOI:** 10.3389/fphar.2021.614586

**Published:** 2021-04-01

**Authors:** Cristina Mondello, Salvatore Roccuzzo, Orazio Malfa, Daniela Sapienza, Patrizia Gualniera, Elvira Ventura Spagnolo, Nunzio Di Nunno, Monica Salerno, Cristoforo Pomara, Alessio Asmundo

**Affiliations:** ^1^ Department of Biomedical and Dental Sciences and Morphofunctional Imaging, University of Messina, Messina, Italy; ^2^ Institute of Legal Medicine and Department of Surgical and Medical Sciences, University “Magna Graecia”, Catanzaro, Italy; ^3^ Section of Legal Medicine, Department of Health Promotion Sciences, Maternal and Infant Care, Internal Medicine and Medical Specialties, University of Palermo, Palermo, Italy; ^4^ Department of History, Society and Studies on Humanity, University of Salento, Lecce, Italy; ^5^ Department of Medical, Surgical and Advanced Technologies “G.F. Ingrassia”, University of Catania, Catania, Italy

**Keywords:** COVID-19, SARS-CoV-2, autopsy, pathological findings, diffuse alveolar damage, immuno-thrombotic microangiopathy, hyperinflammatory state, systematic review

## Abstract

**Introduction:** The World Health Organization declared the COVID-19 pandemic in March 2020. COVID-19 still represents a worldwide health emergency, which causesa severe disease that has led to the death of many patients. The pathophysiological mechanism of SARS-CoV-2 determining the tissue damage is not clear and autopsycan be auseful tool to improve the knowledge of this infection and, thus, it can help achieve a timely diagnosis and develop an appropriate therapy. This is an overview of the main post-mortem findings reporting data on the infection effects on several organs.

**Methods:** A systematic literature search was conducted in the PubMed database searching for articles from 1 January to August 31, 2020. Thearticles were selected identifying words/concepts in the titles and/or abstracts that indicated the analysis of the morphological/pathological tissue injuries related to SARS-CoV-2 disease by several investigations.

**Results:** A total of 63 articles were selected. The main investigated tissue was the lung showing a diffuse alveolar damage (DAD) frequently associated with pulmonary thrombotic microangiopathy. Inflammatory findings and vascular damage were observed in other organs such as heart, liver, kidney, brain, spleen, skin and adrenal gland. The immunohistochemical analysis showed tissue inflammatory cells infiltrates. The virus presence was detected by several investigations such as RT-PCR, immunohistochemistry and electron microscope, showing the effect ofSARS-CoV-2not exclusively in the lung.

**Discussion:** The evidence emerging from this review highlighted the importance of autopsy to provide a fundamental base in the process of understanding the consequences ofSARS-CoV-2 infection. COVID-19 is strictly related to a hyper inflammatory state that seems to start with DAD and immuno-thrombotic microangiopathy. Massive activation of the immune system and microvascular damage might also be responsible for indirect damage to other organs, even if the direct effect of the virus on these tissues cannot be excluded.

## Introduction

In December 2019, a new Coronavirus determined a cluster of infectious diseases in Wuhan, China. In a few months, it spread across the globe, leading the WHO to declare a COVID-19 pandemic on March 11, 2020. This new pathogen is SARS-CoV-2, and the related disease in humans is defined as Coronavirus disease 2019 (COVID-19) by WHO (Weiss and Murdoch, 2020). COVID-19 is still considered a worldwide health emergency, causing pneumonia, severe acute respiratory syndrome (SARS), multiorgan dysfunction, and death among others (Weiss and Murdoch, 2020). The WHO COVID-19 Dashboard, as of September 2, 2020, reported more than 26 million confirmed cases worldwide, of which 852,758 deaths (World Health Organization, 2020).

The primary mode of human-to-human transmission of the virus is close contact, mainly by inhalation of respiratory droplets (Lauer et al., 2020; Osborn et al., 2020). The incubation period ranges between 2 and 11 days, up to a maximum of 14 days (Chen et al., 2020). There are various signs and symptoms of COVID-19. Patients report fever, dry cough, and diarrhea as the most common; other symptoms are represented by myalgia, fatigue, anorexia, nausea and vomiting, confusion, headache, sore throat, rhinorrhoea. In severe cases, dyspnea, ARDS, arrhythmia and acute cardiac injury, acute kidney injury, liver damage at various degrees, and septic shock (Wang D. et al., 2020; Wang et al., 2020b; Huang et al., 2020). It was reported that the median time from the first symptom to dyspnea was 5 days, to hospital admission was 7 days, and to ARDS was 8 days (Wang et al., 2020b).

Evidence highlights that the individual response against SARS-CoV-2 differs due to genetic variations in the human population that may affect the severity of the infection and a better understanding of such variations could help identify the subjects who are most at risk (Singh et al., 2020).

The main tool to identify or confirm the COVID-19 infection is the reverse transcription-PCR (RT-PCR) on respiratory tract specimens such as nose or throat swabs (Corman et al., 2020; Udugama et al., 2020). Moreover, the serological and immunological assays are used to detect the antibodies produced by subjects following virus exposure or the antigenic proteins in infected individuals (Udugama et al., 2020). The molecular test has a shorter “window” period compared to the immunological test, with a positive outcome in the earliest infection phase: a range from 0 to 5 days has been reported to ascertain RNA-virus positivity with nose or throat swabs, or combination of both (Carter et al., 2020; Sessa F. et al., 2020).

The SARS-CoV-2 outbreak has significantly changed the clinical and methodological approaches in all healthcare branches leading to specific recommended protocols that must be implemented for the safety of both patients and practitioners (Meng et al., 2020; Wong et al., 2020). The same concerns are equally applicable in the management of corpses of the people died “from” or “with” COVID-19 or, in general, subjects died during the outbreak. The Centers for disease Control and Prevention (CDC) have published recommendations and safety strategies to adopt during confirmed and suspected COVID-19 autopsies and about the safe management of corpses at the epicenter of the outbreak (CDC, 2020). Furthermore, several scientific societies and research groups proposed documents to help healthcare professionals and morgue staff, providing data and information about possible risks related to corpse management and practical guidance of preventive measures that must be used in suspected, probable, or confirmed COVID-19 deaths (Hanley et al., 2020a; Basso et al., 2020; Fineschi et al., 2020; Yaacoub et al., 2020). However, some countries, like Italy, have chosen not to perform the clinical autopsy, except for selected cases (Pomara et al., 2020; Salerno et al., 2020). In association with epidemiological, clinical, and laboratory data, clinical autopsies may be useful to improve the knowledge on the pathophysiology of COVID-19 infection and, thus, to reach a timely diagnosis and therapy.

This review aims to provide an overview of the main microscopic findings and biomarkers described in literature to perform the diagnosis of death due to COVID-19, highlighting the importance of autoptic examination in ascertaining the cause of death. These data are fundamental in order to distinguish between death “from” and “with” COVID-19.

## Materials and Methods

The review has been conducted by employing the PubMed database, searching for articles from 1 January to August 31, 2020, written in English. The key terms used were “COVID-19” or “SARS-CoV-2” or “nCoV” in association with the terms “AUTOPSY” or “IMMUNOCHEMISTRY” or “IMMUNOHISTOCHEMICAL” or “IMMUNOHISTOCHEMISTRY”. The main rule for article selection was the identification in the titles and/or the abstracts of words/concepts indicating the analysis of the morphological/pathological tissue injuries related to SARS-CoV-2 disease. The article selection was particularly focused on those describing autopsy findings and, thus, on the macroscopic, histological, and immunohistochemical data. The articles were read entirely if the abstract indicated that the paper’s content potentially met the inclusion criteria. Articles were excluded by title, abstract, or full text if not dealing with the topic. Article reviews were also excluded. The works considered relevant were analyzed in-depth, focusing on the gross examination data, histological findings, immunohistochemical results, ultrastructural morphology and molecular diagnosis of SARS-CoV-2 infection. Full-text articles were analyzed, and data were extracted by two authors and reviewed by anothertwo authors.

## Results

**FIGURE 1:**
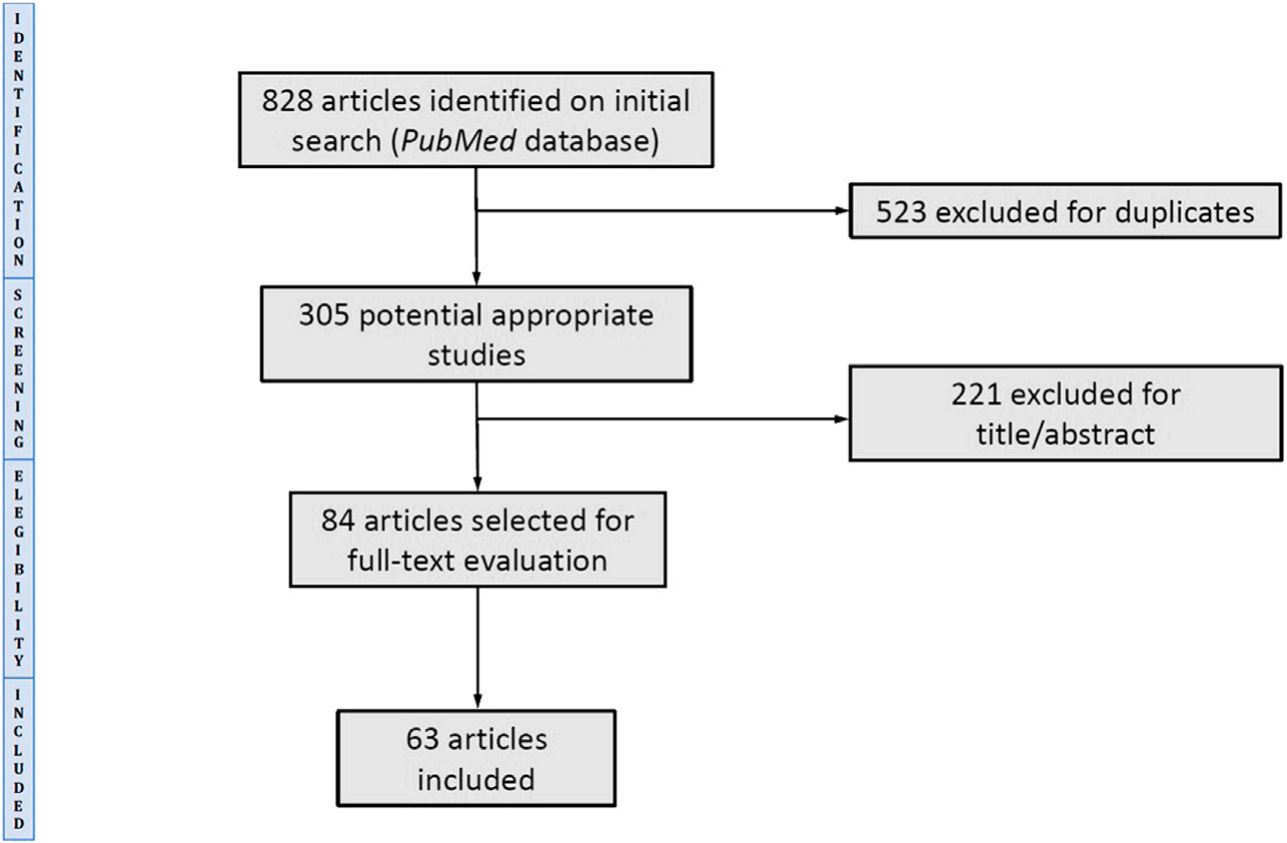
Flow diagram of the article selection procedure.

Figure 1 shows the study selection result. A total of 63 articles were selected for review, reporting the pathological findings related to COVID-19. The articles focused mainly on the evidence from the lungs to describe patterns suggesting the complexity of the pathology. Then, the review showed that researchers also investigated the heart, liver, and kidneys; few articles reported the analysis of other organs or tissues like the adrenal gland, brain, skin, spleen. The investigated papers described evidence resulting from macroscopic and histological findings, immunohistochemical investigations, post-mortem molecular diagnosis of SARS-CoV-2 infection, and electron microscopy analysis.

### Risk of Bias

To reduce the risk of bias two authors independently assessed the included studies. The risk of bias of this systematic review regards data about pre-existing or concomitant pathology resulting from microscopic findings reported by each article. Additionally, it must be noted that RT-PCR data could represent another risk of bias due to their intrinsic and extrinsic limitations (Sessa F. et al., 2020; Carter et al., 2020).

### Lung Findings

The main lung findings reported in each reviewed article are summarized in Table 1. The gross examination often showed heavy, edematous and congested lungs (Aguiar et al., 2020; Konopka et al., 2020b; Bradley et al., 2020; Buja et al., 2020; Carsana et al., 2020; Cipolloni et al., 2020; Craver et al., 2020; Edler et al., 2020; Fitzek et al., 2020; Fox et al., 2020; Heinrich et al., 2020; Lacy et al., 2020; Lax et al., 2020; Magro et al., 2020; Menter et al., 2020; Navarro Conde et al., 2020; Okudela et al., 2020; Oprinca and Muja, 2020; Sekulic et al., 2020; Skok et al., 2020; Suess and Hausmann, 2020; Wichmann et al., 2020; Yan et al., 2020; Youd and Moore, 2020), with reddish-dark areas (Cipolloni et al., 2020; Edler et al., 2020; Fox et al., 2020; Heinrich et al., 2020; Lax et al., 2020; Menter et al., 2020; Remmelink et al., 2020; Salerno et al., 2020; Wichmann et al., 2020). Pulmonary consolidations of different sizes, from patchy to diffuse, were also described (Aguiar et al., 2020; Bradley et al., 2020; Fox et al., 2020; Oprinca and Muja, 2020; Santana et al., 2020; Schaefer et al., 2020; Yan et al., 2020; Youd and Moore, 2020). In some cases, clear hemorrhagic areas were reported (Bradley et al., 2020; Buja et al., 2020; Fox et al., 2020; Lacy et al., 2020; Nunes Duarte-Neto et al., 2020); Cipolloni et al. (Cipolloni et al., 2020) observed whitish fibrotic areas in one case. Some groups of researchers described thromboemboli in large pulmonary arteries and/or small and mid-sized arteries (Hanley et al., 2020b; Buja et al., 2020; Grimes et al., 2020; Remmelink et al., 2020; Skok et al., 2020). In some cases, pleura showed signs of pleurisy with pleural adhesion and effusion (Aguiar et al., 2020; Wang C. et al., 2020; Bradley et al., 2020; Edler et al., 2020; Fox et al., 2020; Navarro Conde et al., 2020; Oprinca and Muja, 2020; Remmelink et al., 2020; Santana et al., 2020; Youd and Moore, 2020).

**TABLE 1:** Lung main data observed in COVID-19 deaths, derived from article enrolled in literature review.

Author(s)	Sample	Gross examination	Microscopic finding(s)	Immunoistochemistry	Post-mortem molecular test	Other	
Okudela et al. (2020)	1 (F, 93 years)	Edematous, heavy, solid and firm, partially glittering, brownish-red, viscous exudate	Mixture of regenerating/desquamative pneumocytes, macrophages and fibroblasts in alveoli; HM; focal alveolar hemorrhage, DAD	N.A.	N.A.	N.A.	
Wichmann et al. (2020)	12 (3 F; 9 M; 52–87 years)	Lung congestion with pale and reddish blue areas; friable consistency	DAD, aPC, mTE, capillary congestion, protein-enriched interstitial edema, SM, small lung arteries microthrombi	N.A.	RT-PCR (positive 12/12)	N.A.	
Heinrich et al. (2020)	1 (M; 59 years)	Edema, deep-red discolorations	DAD, HM, mTE, capillary congestion, protein-enriched interstitial and intra-alveolar edema, mononuclear inflammatory cells infiltration, hPC	CD8	RT-PCR (positive)	N.A.	
Bradley et al. (2020)	14 (7 M, 7 F; 42–84 years)	Edema, intraparenchymal hemorrhage, pulmonary consolidation, pleural fluid	DAD, intra-alveolar fibrin, HM, or loosely organizing connective tissue in the alveolar septal wall, multinucleated cells, chronic interstitial inflammation, perivascular lymphocytic inflammation, focal mT	TTF-1 (positive); CD68 (negative); SARS-cov-2 sP (positive 4/4)	RT-PCR (positive)	TEM: Viral particles in pneumocytes (1/14), viral particles either outside cells in close proximity to the cell membrane or inside the cells in aggregates confined within vesicles	
Aguiar et al. (2020)	1 (F; 31 years)	Heavy and rubbery, hemorrhagic edema, whitish consolidation, pleural effusion	DAD,HM, fibrin in alveoli, hPC, intraalveolar macrophages, interstitial edema. Lympho-monocytic infiltrates	CD68, CD3, CD20	RT-PCR (tracheobronchial swab positive)	N.A.	

Sekulic et al. (2020)	2 (2 M,; 54 and 81 years)	Heavy, firmness, congested	DAD, HM, scattered SM, hPC, intra-alveolar fibroblastic, proliferation, interstitial edema, multinucleated giant cells in alveoli	N.A.	RT-PCR (positive 2/2)	N.A.	
Cipolloni et al. (2020)	2 (M; 42 and 70 years)	Heavy edematous with pink froth, dark and reddish areas (1/2), whitish fibrotic areas (1/2)	Severe organizing pneumoniae, plasma cells, macrophages and lymphocytes in alveoli, proliferation of connective tissue of alveolar septa with leukocytes infiltration, fibrin deposition in vessel lumens and walls, modest fibrin perivasal deposit (1/2); DAD, HM, hPC, protein and fibrinous exudate in alveoli, macrophage alveolar infiltrate (1/2)	CD4, CD8, CD20, CD68, CD79, factor VIII, TNFα, IL-6, ACE2, SARS-COV-2 NP (positive)	RT-PCR (nasopharyngeal and oropharyngeal swabs negative 1/2); nasopharyngeal swab positive 1/2)	N.A.	
Magro et al. (2020)	5 (2 F, 3 M; 32–73 years)	Congested, hemorrhagic	Thrombotic necrotizing capillary injury (1/5); septal capillaries luminal and mural fibrin deposition, focal intra-alveolar neutrophils and monocytes, intra-alveolar fibrin deposition, focal HM, hPC	C4d, C3d, C5b-9, MASP2 (positive); SARS-COV-2 sP and eP (positive 1/5)	N.A.	N.A.	
Edler et al. (2020)	80 (33 F, 47 M; 52–96 years)	Heavy and edematous, signs of pleurisy, solidified but also fragile, dark-red	DAD, aPC, fibroblasts, protein-rich exudate, HM, SM, fibrosis, lymphocytes and plasma cell infiltrates in small pulmonary arteries (8/12); granulocyte-dominated focal confluent bronchopneumonia (4/12)	N.A.	RT-PCR (nasopharyngeal swabs positive)	N.A.	
Suess and Hausmann (2020)	1 (M; 59 years)	Edematous, firm and rubbery, dark-red, pleural surface hemorrhage, pleurisy singns	DAD, HM, proteinaceous exudates, alveolar hemorrhage, intra-alveolar fibrin deposition and macrophages, pulmonary capillaries mT, some fresh thrombi in pulmonary arteries	TTF-1, CD68	N.A.	N.A.	
Navarro Conde et al. (2020)	1 (M, 69 years)	Deep red, increased weight and density, pleural adhesions	Edema and intraalveolar hemorrhage, DAD, desquamation of type II pneumocytes,HM, and thrombi in the medium sized vessels, abundant intraalveolar macrophages and occasional multinucleated giant cells, hPC, myofibroblastic proliferations, SM, mild lymphoid infiltrate with abundant macrophages	Cytokeratin AE1/AE3, TTF-1, CD68	N.A.	N.A.	
Carsana et al. (2020)	38 (33 M; 5F; 32–86 years)	Heavy, congested, edematous	DAD, capillary congestion, necrosis of pneumocytes, HM, interstitial and intra-alveolar edema hPC, SM with atypia, platelet–fibrin thrombi, macrophages in alveoli, lymphocytes in interstitium	CD68; CD3; CD45; CD61; TTF1; p40; Ki-67	N.A.	TEM: Viral particles predominantly in pneumocytes	
Youd and Moore (2020)	3 (N.A.)	Edematous, consolidation, plural adhesions	DAD, HM, hPC, clumps of fibrin within alveoli, widening of alveolar walls and interstitium with lymphocytic infiltrate	N.A.	RT-PCR (lung swab positive 1/9)	N.A.	
Craver et al. (2020)	1 (M, 17 years)	Heavy, congested	Congestion, focal acute hemorrhage, edema, slightly thickened bronchial basement membranes and mild chronic inflammation with only occasional eosinophils	N.A.	RT-PCR (nasopharyngeal swabs positive)	N.A.	
Lacy et al. (2020)	1 (F, 58 years)	Moderately heavy and edematous, firm areas of hemorrhage	DAD with diffuse proteinaceous edema and dense amphophilic concretions along alveolar septae, HM, mild mononuclear infiltrates, hPC, focal multinucleated cells with bizarre forms, acute alveolar hemorrhage, alveolar macrophages, alveolar fibrin	N.A.	RT-PCR (main bronchi swabs positive)	N.A.	
Yan et al. (2020)	1 (F, 44 years)	Heavy, evidence of pleuritis, pleuric erythema overlying regions of consolidation, pulmonary edema, areas of dense consolidation	Edema, isolated areas of infarction, DAD, diffuse interstitial lymphocytic infiltrates and fibrinous exudates, HM in patchy areas, extensive desquamation of pneumocytes, multinucleated giant cells, hPC, extensive and widespread perivascular lymphocytic cuffing, few foci of lymphocytic infiltration in vessel walls	N.A.	N.A.	TEM: Viral like-particles in cytoplasmic vesicles of reactive pneumocytes and free within extracellular alveolar spaces; numerous fibrin aggregates in blood vessels	
Konopka et al. (2020b)	1 (M; 37 years)	Heavy, mucus in airways, consolidations	DAD, edema, focal hPC, scattered HM, rare fibrin-thrombi in small vessels, patchy fibrinous exudate with mononuclear inflammatory cells and scattered neutrophils in airspaces	N.A.	N.A.	N.A.	
Menter et al. (2020)	21 (4 F; 17 M; 53–96 years))	Heavy and firm, bluish–red, congested, areas of consolidation, suppurative bronchopneumonia	Capillary congestion, DAD, reactive pneumocytes and syncytial cells, alveolar capillaries mT, bronchopneumonia, pulmonary embolism (4/21), lymphoid infiltrates, hemorrhage, vasculitis (1/21)	Fibrin, ATTR, CD3,CD4, CD8, CD20, CD68, TTF1	RT-PCR (formalin-fixed paraffin-embedded tissue positive)	N.A.	
Fox et al. (2020)	10 (F and M; 44–78Y)	Edematous, firm, dark-coloured hemorrhage	DAD, edema, HM, interstitial lymphocyte infiltrates surrounding the larger bronchioles and blood vessels, platelets and mT in small vessels, foci of hemorrhage, desquamated type 2-pneumocytes, thickened alveolar capillaries, fibrin-thrombi aggregate to inflammatory cells in capillaries and small vessels, megakaryocytes in alveolar capillaries	CD4, CD8, CD68, TTF-1	RT-PCR (tracheobronchial swabs positive)	TEM: Infected pneumocyte	
Lax et al. (2020)	11 (3 F; 8 M 66–91 years)	Congested, thrombotic material in pulmonary arteries branches (11/11), pulmonary infarctions (3/11)	DAD, edema, HM, pneumocytes and fibroblasts proliferation, pulmonary artery thrombosis (11/11), infarction (9/11), pneumonia	N.A.	N.A.	N.A.	
Buja et al. (2020)	3 (M; 34–62 years)	Congestion, multiple bilateral segmental pulmonary thromboemboli, multiple areas of hemorrhage	DAD, HM, aPC, macrophages in alveoli, alveolar SM, fibrin deposits in alveoli, multiple mTE with areas of pulmonary hemorrhage and infarction, lymphocytic pneumonitis, intra-alveolar fibrinous exudate, intra-capillary megakaryocytes	CD3, CD61, CD68, TTF-1, CK-7	RT-PCR (nasopharyngeal swabs positive 2/3)	N.A.	
Oprinca and Muja (2020)	3 (1 F 79 years; 1 F 27 years; 1 M 70 years)	Edematous, congested, areas of consolidation, pleural effusion	DAD, HM, hPC, vasculitic reaction, mT in small vessels (2/3), lymphocytic and neutrophilic infiltrates	Pancytokeratin CK- AE1–AE3, CK-MNF116, CD3, CD5, CD20 (positive). CD23 and CD30 (negative)	RT-PCR (positive)	N.A.	
Fitzek et al. (2020)	1 (M, 59 years)	Edema, dark red, increased consistence, greyish-yellow multifocal areas	DAD, HM, mT	N.A.	RT-PCR (nasopharyngeal and lung swabs positive)	N.A.	
Skok et al. (2020)	19 (7M, 12F; 66–93 years)	Congested, thrombi in small and mid-sized arteries	Edema, HM, pulmonary artery thrombosis, infarction, bronchopneumonia, fibrosis	N.A.	RT-PCR (positive)	N.A.	
Remmelink et al. (2020)	17 (12M, 5F; 62–77 years)	Heavy, firm, dark/red areas of hemorrhage, thrombi in large pulmonary arteries (2/17), infarction (4/17)	DAD, mT, infarct (4/17), bronchopneumonia	Sars-CoV-2 NP (positive 11/17)	RT-PCR (positive 16/17)	N.A.	
Santana et al. (2020)	1 (M, 71 years)	Focal areas of consolidation in the right lower lobe (aspergillus)	Neutrophilic infiltrates in alveoli, fibrin-thrombi in medium-sized artery, SM, bronchopneumonia, (aspergillus: Hyphae and fungal spores, microscopic cavitation)	N.A.	N.A.	N.A.	
Schaefer et al. (2020)	7 (2 F, 5 M; 50–77 years)	Patchy consolidation	DAD, HM, interstitial-peribronchial lymphocytes, intraalveolar macrophages, hPC, pulmonary embolism, mT	Sars–CoV-2 NP (positive 5/7), TTF-1, PU.1, p63, MUC5AC, FOXJ1	N.A.	N.A.	
Nunes Duarte-Neto et al. (2020)	10 (5 F; 5 M; 33–83 years)	N.A. (biopsy)	DAD, SM, lymphocytic inflammation, arteriolar mT (8/10), alveolar megakaryocytes, alveolar hemorrhage, pneumonia	CD4, CD8, CD20, CD57, CD68, TTF-1, p63, Ki67	RT-PCR (positive 6/7)	N.A.	
Grimes et al. (2020)	2 (M, n.a.)	Thromboembolism with main pulmonary arteries occlusion, deep venous thrombosis, pulmonary consolidation	Pink and red areas (line of zhan) in thromboemboli with alternating strata of platelets and fibrin with admixed layers of erythrocytes	N.A.	N.A.	N.A.	
Hanley et al. (2020a)	10 (7 M, 3 F; 73 years median age)thereof 1 MIA biopsy	Subpleural petechial hemorrhage, pulmonary thromboemboli	DAD, mTE, macrophage and lymphocyte inflammation	CD3, CD4, CD20, CD34, CD56, CD68-PGM1, CD61	RT-PCR (positive 5/5)	N.A.	
Wang C. et al. (2020)	2 (1 F, 53 years; 1 M, 62 years)	Hepatization, pleural effusion, fibrotic pleural adhesion	DAD, HM, macrophages with scattered neutrophils and lymphocytes, serous and fibrinoid exudate in alveoli, focal or patchy hemorrhage, peribronchiolar metaplasia, type II pneumocytes proliferation, mT in small veins and arteries	IL-6, IL-10, TNFa, PD-L1, CD20, ACE2, CD3, CD4, CD8, CD68	RT-PCR (paraffin-embedded lung positive)	N.A.	
Aiolfi et al. (2020)	2 (M; 56 and 70 years)	N.A. (biopsy)	DAD, edema, desquamation of pneumocytes, inflammatory cells in alveoli and interstitial tissue, intravascular hemorrhagic thrombosis	TTF-1, NapsineA, CD3, CD34, CD5, CD8, CD31, CK7, and collagen type IV	N.A.	N.A.	
Martines et al. (2020)	8 (4 F, 4 M; age n.a.)	N.A.	DAD, edema, HM, hPC, desquamation of pneumocytes, fibrin deposits, alveolar infiltrates, increased macrophages	Sars-CoV-2 NP (positive)	RT-PCR (positive)	TEM: Intracellular virions in type II pneumocytes and in cytoplasmic vesicles or phagosomes of alveolar macrophages, viral particles also associated with fibrin or hyaline membranes in alveoli	
Xu et al. (2020)	1 (M; 50 years)	N.A. (biopsy)	DAD, HM, edema, cellular fibromyxoid exudates, desquamative pneumocytes, interstitial mononuclear infiltrate, multinucleated syncytial cells	N.A.	N.A.	N.A.	
Tian et al. (2020a)	2 (1 F, 84 years; 1 M, 73 years)	N.A. (biopsy)	DAD, edema, hPC, proteinaceous exudates, interstitial thickening, intra-alveolar fibrin, inflammatory cells infiltrates in alveoli, fibroblastic proliferation	N.A.	N.A.	N.A.	
Tian et al. (2020b)	4 (1 F, 3 M; 59–81 years)	N.A. (biopsy)	DAD, HM, fibrin exudates, hPC, alveolar walls thickening with mononuclear inflammatory cells infiltrates	N.A.	RT-PCR (positive 1/4)	N.A.	
Karami et al. (2020)	1 (F; 27 years; pregnant)	N.A.	HM, pneumocyte proliferation, metaplastic changes, lymphocytes and macrophages infiltrates	N.A.	RT-PCR (positive)	N.A.	
Schaller et al. (2020)	10 (3 F, 7 M; 64–90 years)	N.A.	DAD, edema, HM, hPC, exudate, thickened alveolar septa, perivascular plasma cells infiltration, fibroblastic proliferation	N.A.	RT-PCR (nasopharyngeal, tracheal, bronchial swabs and pleural effusion positive)	N.A.	
Cai et al. (2020)	7 (2 F, 5 M; 56–68 years)	N.A.	Interstitial inflammation, thickened alveolar septum, fibrous connective tissue proliferation, mT (1/7), septal capillary damage (2/7)	N.A.	N.A.	N.A.	
von Weyhern et al. (2020)	6 (2 F, 4 M; 58–82 years)	N.A.	DAD, edema, hPC, polymorphous pneumocytes, organizing pneumonia pattern	CD3	N.A.	N.A.	
Shao et al. (2020)	1 (M, 65 years)	N.A. (biopsy)	DAD, edema, MH, hPC, intra-alveolar fibrinous exudates, inflammatory cells in alveoli, interstitial fibrosis, mT in pulmonary capillaries	TTF-1	N.A.	N.A.	
Dolhnikoff et al. (2020)	10 (5 M; 5 F; 33–83 years)	N.A. (biopsy)	DAD, lymphocytic infiltration, fibrin-thrombi in small pulmonary arterioles (8/10), endothelial tumefaction and large number of pulmonary megakaryocytes in the pulmonary capillaries, alveolar hemorrhage foci	N.A.	N.A.	N.A.	
Konopka et al. (2020a)	8 (M, 37 years; M, 46 years; F, 79 years; F, 63; F, 49 years; M, 44 years; M, 55 years; M, 67 years)	N.A.	DAD, HM, fibrin-thrombi in small precapillary vessels and/or muscular arteries (5/8), airspace organization, AFOP-like intra-alveolar fibrin (5/8), perivascular inflammation/endothelialitis (6/8), acute bronchopneumonia	N.A.	RT-PCR (nasopharyngeal swabs positive)	N.A.	
Ackermann et al. (2020)	7 (2 F; 5 M; 58.8–91.5 years)	N.A.	DAD, necrosis of alveolar lining cells, hPC, linear intra-alveolar fibrin deposition	CD3, CD4, CD8, CD15, CD20, CD68, CD61, ACE2, TMPRSS2, fibrinogen, podoplanin	N.A.	TEM: Endothelium ultrastructural damage and intracellular SARS-COV-2; intussusceptive angiogenic and/or conventional sprouting angiogenesis	
Wang et al. (2020b)	2 (1 F, 79 years; 1 M, 50 years)	N.A. (biopsy)	DAD, HM, desquamation of pneumocytes	N.A.	Sars-CoV-2 (positive)	N.A.	
Prilutskiy et al. (2020)	4 (1 F; 3 M; 64–91 years)	N.A.	DAD	CD163, Pax-5, CD3	N.A.	N.A.	
Barton et al. (2020)	2 (M; 47–72 years)	N.A.	DAD, HM, interstitial lymphocytic infiltrates, mT in few small pulmonary artery branches, alveolar septal capillaries congestion, edema fluid in airspaces, mild chronic inflammation in bronchi and bronchioles	CD3, CD4, CD8, CD20, CD68	N.A.	N.A.	
Zhang et al. (2020)	1 (M; 72 years)	N.A. (biopsy)	DAD, hPC, alveolar fibrinous exudates, interstitial fibrosis, chronic inflammatory infiltrates, fibrous plugs and fibrin in alveoli	Sars–CoV-2 NP (positive)	N.A.	N.A.	
Wang F. et al. (2020)	2 (F, 94 years and male 65 years)	N.A. (biopsy)	Alveolar exudates, HM, hPC, widened alveolar septa and few lymphocytic infiltrate, fibrosis, neutrophilic infiltrates	CD3, CD4, CD8, CD21, CD10 (positive)	N.A.	ISH negative	
Rapkiewicz et al. (2020)	7 (4F, 3M; 44.65 years)	Congested	DAD, HM, pneumocytes desquamation, platelet-rich mT, sparse septal and perivascular lymphocytic infiltrates, bronchopneumonia focally necrotizing	Sars-CoV-2 NP (n.a.), CD3, CD4, CD8, CD61 (positive)	N.A.	TEM: Rare virions	
Kantonen et al. (2020)	4 (3M, 1F; 63–90 years)	N.A.	DAD, mT	N.A.	RT-PCR (positive)	N.A.	
Varga et al. (2020)	3 (M, 71 years; F, 58 years) + M, 69 years (small intestine resection)	N.A.	Inflammatory cells associated with endothelium, mononuclear cells, small lung vessels congested	N.A.	N.A.	N.A.	

N.A., not available; DAD, diffuse alveolar damage; HM, hyaline membrane; aPC, activated type II pneumocytes; hPC, hyperplasic type II pneumocytes; mTE, microvascular thromboemboli; mT, microthrombi; SM, squamous metaplasia; Sars-CoV-2 NP, Sars-CoV-2 nucleo-capsid protein; Sars-CoV-2 sP, Sars-CoV-2 spike protein; Sars-CoV-2 eP, Sars-CoV-2 envelope protein; ATTR, amyloid transthyretin; TTF1, thyroid transcription factor 1; ACE2:angiotensin-converting enzyme 2, MASP2, Mannan Binding Lectin Serine peptidase 2; PGM1, Phosphoglucomutase-1; PD-L1l, Death-Ligand 1; TMPRSS2, Transmembrane Serine protease 2; ISH, *in situ* hybridization; TEM, transmission electron microscopy.

The most encountered histological finding was diffuse alveolar damage (DAD) at different stages, mainly in exudative and proliferative phases, characterized by hyaline membranes, intra-alveolar and/or interstitial edema also proteinaceus, intra-alveolar fibrinous exudate, intra-alveolar mononuclear cells infiltrates (macrophages, lymphocytes, neutrophils), type 2 pneumocyte hyperplasia/activation, squamous metaplasia (Okudela et al., 2020; Wichmann et al., 2020; Heinrich et al., 2020; Bradley et al., 2020; Aguiar et al., 2020; Sekulic et al., 2020; Cipolloni et al., 2020; Edler et al., 2020; Suess and Hausmann, 2020; Navarro Conde et al., 2020; Carsana et al., 2020; Youd and Moore, 2020; Lacy et al., 2020; Yan et al., 2020; Konopka et al., 2020b; Menter et al., 2020; Fox et al., 2020; Buja et al., 2020; Oprinca and Muja, 2020; Fitzek et al., 2020; Skok et al., 2020; Remmelink et al., 2020; Santana et al., 2020; Schaefer et al., 2020; Nunes Duarte-Neto et al., 2020; Grimes et al., 2020; Hanley et al., 2020b; Wang C. et al., 2020; Aiolfi et al., 2020; Martines et al., 2020; Xu et al., 2020; Tian et al., 2020a; Tian et al., 2020b; Karami et al., 2020; Schaller et al., 2020; Cai et al., 2020; von Weyhern et al., 2020; Shao et al., 2020; Dolhnikoff et al., 2020; Konopka et al., 2020a; Ackermann et al., 2020; Wang et al., 2020a; Prilutskiy et al., 2020; Barton et al., 2020; Zhang et al., 2020; Wang X.-X. et al., 2020; Rapkiewicz et al., 2020; Kantonen et al., 2020). In a few cases, alveolar hemorrhage was observed (Buja et al., 2020; Craver et al., 2020; Dolhnikoff et al., 2020; Grimes et al., 2020; Lacy et al., 2020; Okudela et al., 2020; Suess and Hausmann, 2020). Pneumonia or bronchopneumonia pictures were also described as focal or diffuse (Okudela et al., 2020; Cipolloni et al., 2020; Edler et al., 2020; Menter et al., 2020; Lax et al., 2020; Buja et al., 2020; Remmelink et al., 2020; Santana et al., 2020; Nunes Duarte-Neto et al., 2020; von Weyhern et al., 2020; Konopka et al., 2020a; Rapkiewicz et al., 2020). In several cases, the presence of fibrin-enriched thrombi in vessels was reported, mostly appearing as microthrombi in alveolar capillaries and/or in small vessels (Konopka et al., 2020a; Barton et al., 2020; Konopka et al., 2020b; Bradley et al., 2020; Buja et al., 2020; Cai et al., 2020; Carsana et al., 2020; Dolhnikoff et al., 2020; Fitzek et al., 2020; Fox et al., 2020; Grimes et al., 2020; Kantonen et al., 2020; Menter et al., 2020; Navarro Conde et al., 2020; Nunes Duarte-Neto et al., 2020; Okudela et al., 2020; Oprinca and Muja, 2020; Rapkiewicz et al., 2020; Remmelink et al., 2020; Santana et al., 2020; Schaefer et al., 2020; Shao et al., 2020; Skok et al., 2020; Suess and Hausmann, 2020; Wichmann et al., 2020). Aiolfi et al. (2020) found massive intravascular hemorrhagic thrombosis of peripheral vessels associated with diffused endothelial hyperplasia and general thickening of the muscular wall. Moreover, damage of small vessels was reported as thrombotic necrotizing capillary injury (Magro et al., 2020), infiltrate of lymphocytes and plasma cells (Edler et al., 2020), vasculitis (Menter et al., 2020; Oprinca and Muja, 2020), septal capillary damage (Cai et al., 2020), endothelial tumefaction with a large number of pulmonary megakaryocyte in capillaries (Dolhnikoff et al., 2020), perivascular inflammation/endothelialitis (Ackermann et al., 2020; Wang et al., 2020a; Varga et al., 2020). Additionally, Ackermann et al. (2020) described fibrin thrombi in arterioles associated with intussusceptive angiogenesis.

Several immunohistochemical markers were used to identify better the inflammatory cells infiltrates. T and B cells were investigated by CD3, CD5, CD4 (T helper cells), CD8 (cytotoxic T cells) and CD20 (B lymphocytes) antibodies **(** Heinrich et al., 2020; Aguiar et al., 2020; Cipolloni et al., 2020; Carsana et al., 2020; Menter et al., 2020; Fox et al., 2020; Buja et al., 2020; Oprinca and Muja, 2020; Nunes Duarte-Neto et al., 2020; Grimes et al., 2020; Hanley et al., 2020b; Aiolfi et al., 2020; von Weyhern et al., 2020; Ackermann et al., 2020; Barton et al., 2020; Wang X.-X. et al., 2020; Rapkiewicz et al., 2020; Varga et al., 2020); a study reported the analysis of CD57 ^+^ showing the presence of sparse Natural Killer cells not varying according to DAD pattern (Nunes Duarte-Neto et al., 2020). Macrophages, analyzed using CD68, were mostly sited in alveolar spaces and in fibroproliferative areas (Ackermann et al., 2020; Aguiar et al., 2020; Barton et al., 2020; Buja et al., 2020; Carsana et al., 2020; Cipolloni et al., 2020; Fox et al., 2020; Grimes et al., 2020; Menter et al., 2020; Navarro Conde et al., 2020; Nunes Duarte-Neto et al., 2020; Suess and Hausmann, 2020). CD61 was used to analyze thromboemboli (Ackermann et al., 2020; Hanley et al., 2020b; Buja et al., 2020; Carsana et al., 2020; Rapkiewicz et al., 2020). Magro et al. (Magro et al., 2020) analyzed the complement components C4d and C3d, the terminal complex C5b-9 or membrane attack complex (MAC), and MASP-2 observing the deposition of MAC within the lung septal microvasculature also in normal-appearing lung. As for the routine histology data on type II pneumocytes, TTF-1 (thyroid transcription factor-1) was used to evaluate the involvement of such cells, which appeared, in some cases, enlarged, hyperplastic and atypical with nucleoli viral cytopathic-like changes and many mitotic figures (Aiolfi et al., 2020; Buja et al., 2020; Carsana et al., 2020; Fox et al., 2020; Menter et al., 2020; Navarro Conde et al., 2020; Nunes Duarte-Neto et al., 2020; Schaefer et al., 2020; Shao et al., 2020; Suess and Hausmann, 2020). Angiotensin-converting-enzyme-2 (ACE-2) was investigated as a receptor for host cell entry of SARS-CoV-2, found positive in alveolar epithelial, endothelial cells, alveolar macrophages, and lymphocytes in lung tissue samples (Ackermann et al., 2020; Cipolloni et al., 2020; Grimes et al., 2020). Some researchers carried out immunohistochemistry for virus detection using specific antibodies for nucleocapsid protein (NP) or spike and envelope proteins of SARS-CoV-2, whose positivity was observed in pneumocytes, alveolar macrophages, intralveolar septa, and septal capillary (Bradley et al., 2020; Magro et al., 2020; Martines et al., 2020; Rapkiewicz et al., 2020; Remmelink et al., 2020; Schaefer et al., 2020; Zhang et al., 2020). Notably, Schaefer et al. (2020) reported negative immunostaining in two cases out of total of seven.

Some authors performed the molecular diagnosis of COVID-19 infection using RT-PCR performed by nasopharyngeal, oropharyngeal or tracheobronchial swabs (Aguiar et al., 2020; Konopka et al., 2020a; Buja et al., 2020; Cipolloni et al., 2020; Edler et al., 2020; Wang et al., 2020a; Lacy et al., 2020; Menter et al., 2020; Schaller et al., 2020; Skok et al., 2020), or analyzing lung tissue sampled during autopsy (Bradley et al., 2020; Tian et al., 2020b; Grimes et al., 2020; Heinrich et al., 2020; Kantonen et al., 2020; Lacy et al., 2020; Martines et al., 2020; Nunes Duarte-Neto et al., 2020; Oprinca and Muja, 2020; Wichmann et al., 2020).

Finally, the lung damage was investigated by electron microscopy showing viral particles predominantly located in pneumocytes (Bradley et al., 2020; Carsana et al., 2020; Fox et al., 2020; Yan et al., 2020), free in alveolar space (Yan et al., 2020), in phagosomes of alveolar macrophages (Martines et al., 2020), confirming the above reported immunohistochemical findings; moreover, the viral particles were observed either outside or inside the cells in aggregates confined within vesicles (Bradley et al., 2020). Interestingly, Ackermann et al. (Ackermann et al., 2020) described distorted lung vascularity with structurally deformed capillaries which appeared elongated, with changes in caliber and intussusceptive pillars, and endothelium ultrastructural damage.

### Cardiac Findings

The gross examination of the heart showed myocardial ventricular hypertrophy and dilatation, mainly of the right cavity, in a considerable number of cases (Hanley et al., 2020b; Buja et al., 2020; Fox et al., 2020; Lax et al., 2020; Menter et al., 2020; Oprinca and Muja, 2020; Youd and Moore, 2020). Acute right coronary artery thrombosis was observed in one case (Hanley et al., 2020b). The most frequent microscopic findings included cardiomyocyte hypertrophy (Wang X.-X. et al., 2020; Lacy et al., 2020; Lax et al., 2020; Menter et al., 2020; Nunes Duarte-Neto et al., 2020; Yan et al., 2020), myocardial fibrosis (Craver et al., 2020; Wang X.-X. et al., 2020; Escher et al., 2020; Lax et al., 2020; Nunes Duarte-Neto et al., 2020; Remmelink et al., 2020), focal lymphocytic infiltrate (Buja et al., 2020; Lax et al., 2020; Lindner et al., 2020; Oprinca and Muja, 2020; Rapkiewicz et al., 2020; Schaller et al., 2020), individual cardiomyocyte injury (Buja et al., 2020; Lindner et al., 2020), interstitial edema (Wang X.-X. et al., 2020; Lindner et al., 2020; Nunes Duarte-Neto et al., 2020; Oprinca and Muja, 2020; Yan et al., 2020), acute or previous myocardial infarction (Menter et al., 2020; Nunes Duarte-Neto et al., 2020; Remmelink et al., 2020), coronary artery atheroma and/or atherosclerosis (Lacy et al., 2020; Youd and Moore, 2020). Other rare but significant histopathological changes included amyloidosis (Lax et al., 2020; Menter et al., 2020), coronary small vessel disease (Lax et al., 2020), fibrin microthrombi (Hanley et al., 2020b; Nunes Duarte-Neto et al., 2020; Oprinca and Muja, 2020), thrombosis of myocardial veins (Lax et al., 2020; Rapkiewicz et al., 2020), endocardial thrombi in the left ventricle (Lax et al., 2020), lymphocytic myocarditis/epicarditis/pericarditis (Hanley et al., 2020b; Buja et al., 2020; Rapkiewicz et al., 2020).

Moreover, Tavazzi et al. (2020) examined samples of cardiac tissue using TEM, which revealed the presence of a small group of viral particles or single particles within the damaged interstitial cells of the myocardium, also showed loss of plasmalemma integrity. Lindner et al. (Lindner et al., 2020) clearly described the presence of SARS-CoV-2 RNA in interstitial cells, and macrophage infiltrates by *in situ* hybridization (ISH) performed on paraffin-embedded left ventricle samples.

The myocardial data described in the selected articles are summarized in Table 2.

**TABLE 2:** Summary of the main findings described in reviewed articles on heart in Sars-CoV-2 related death.

Author(s)	Sample	Gross examination	Microscopic finding(s)	Immunoistochemistry	Post-mortem molecular test	Other(s)	
Youd and Moore (2020)	3 (F, 88 years; M, 86 years; F, 73 years)	Enlarged heart (2/3), coronary artery atheroma (3/3), old myocardial scarring (1/3)	Contraction band necrosis (1/3), chronic ischemic changes (2/3)	N.A.	N.A.	N.A.	
Craver et al. (2020)	1 (M, 17 years)	Soft, rubbery, mottled parenchyma	Right and left ventricles diffuse inflammatory infiltrates composed of lymphocytes, macrophages, with prominent eosinophils; foci of myocyte necrosis and minimal interstitial fibrosis	N.A.	N.A.	N.A.	
Lacy et al. (2020)	1 (F, 58 years)	Firm texture, red-brown, moderate coronary atherosclerosis	Minimal mononuclear myocardial inflammatory, myocyte hypertrophy with interstitial and perivascular fibrous tissue	N.A.	N.A.	N.A.	
Yan et al. (2020)	1 (F, 44 years)	Streaking of the right atrial wall, dilated right ventricular chamber	Mild myxoid edema, mild myocyte hypertrophy, focal nuclear pyknosis, rare foci with few scattered lymphocytes in the left ventricular papillary muscle	CD45 (positive)	N.A.	N.A.	
Menter et al. (2020)	21 (4 F; 17 M; 53–96 years)	Myocardial hypertrophy (15/21)	Senile amyloidosis (6/21), peracute myocardial cell necrosis (3/21), acute myocardial infarction (1/21)	(ATTR) (positive)	Variable	N.A.	
Fox et al. (2020)	9 (F and M)	Cardiomegaly and right ventricular dilatation	Scattered individual cell myocyte necrosis, rare areas of lymphocytes adjacent to degenerating myocytes	N.A.	N.A.	N.A.	
Lax et al. (2020)	11 (8 m; 3 F; 66–91 years)	Hypertrophy of both ventricles	Myocardial hypertrophy (11/11), coronary small vessel disease (6/11), myocardial fibrosis (10/11), focal lymphocytic infiltrate (1/11), amyloidosis (1/11), thrombosis of a myocardial vein (1/11), endocardial thrombi in left ventricle (1/11)	N.A.	N.A.	N.A.	

Buja et al. (2020)	23 (7 F, 12 M; 4 unspecified; 34–76 years plus 2 unspecified)	Cardiomegaly (13/23)	Individual cardiomyocyte injury (8/23), lymphocytic epicarditis/pericarditis (3/23), lymphocytic myocarditis (1/23)	CD3, CD68 (1/3 patchy positive)	N.A.	N.A.	
Oprinca and Muja (2020)	3 (1 F 79 years; F 27 years; 1 M 70 years)	Right (3/3) and/or left (2/3) atrial and ventricular dilation and hypertrophy, coronary atheroslerosis	Interstitial edema, vascular congestion (3/3), small number of scattered lymphocytes between the myocardial fibers (1/3), small vessel thrombosis (1/3), contraction of band-like lesions (2/3)	N.A.	N.A.	N.A.	
Remmelink et al. (2020)	17 (12 M, 5 F; 62–77 years)	Cardiomegaly (14/17)	Ischemic cardiomyopathy (15/17), acute myocardial infarction (2/17), cardiac fibrosis (5/17)	N.A.	RT-PCR (14/17 positive)	N.A.	
Nunes Duarte-Neto et al. (2020)	10 (5 F; 5 M; 33–83 years)	N.A. (biopsy)	Cardiomyocytes hypertrophy (9/10), myocardial fibrosis (9/10), previous myocardial infarction (4/10), interstitial edema (9/10), myocarditis (2/10), fibrin thrombi (2/10)	N.A.	N.A.	N.A.	

Hanley et al. (2020a)	10 (7 M, 3 F; 73 years median age) thereof 1 MIA biopsy	Left ventricular hypertrophy, pericarditis (2/9), acute coronary thrombosis in the right coronary artery (1/9)	Thrombi in the microcirculation of the heart (5/9); acute myocardial ischemic damage, pericarditis (2/9), amyloidosis (1/9)	N.A.	RT-PCR (positive 3/5)	N.A.	
Xu et al. (2020)	1 (M; 50 years)	N.A. (biopsy)	Few interstitial mononuclear inflammatory infiltrates	N.A.	N.A.	N.A.	
Schaller et al. (2020)	10 (3 F, 7 M; 64–90 years)	N.A.	Mild lymphocytic myocarditis (1/10), signs of epicarditis (1/10)	N.A.	N.A.	N.A.	
Wang X.-X. et al. (2020)	1 (F, 75 years)	N.A. (biopsy)	Hypertrophic myocytes, fatty infiltration, nuclear pyknosis, interstitial edema, and fibrosis	CD3, CD4, CD8, CD10, CD21 (negative)	N.A.	N.A.	
Rapkiewicz et al. (2020)	7 (4 F, 3 M; 44.65 years)	N.A.	Fibrin microthrombi, limphocyte inflitate (1/7), venous thrombosis (2/7)	CD3, CD4, CD8, CD20, CD61 (positive); C4d (negative)	N.A.	N.A.	
Escher et al. (2020)	5 (M, 22 years; M, 40 years; F, 60 years; M, 25 years; M, 55 years)	N.A. (biopsy)	Active myocarditis (1/5), myocytes necrosis, perivascular fibrosis (4/5)	CD3, CD11a, CD11b, CD45R0+ (positive 4/5); CD54/icam-1 (positive)	RT-qPCR (positive)	N.A.	
Lindner et al. (2020)	39 (23 F, 16 M; 73–89 years)	N.A.	Interstitial edema, lymphocyte and macrophage infiltrates, cardiomyocyte injury	CD3, CD45R0, CD68 (positive)	RT-PCR (positive 24/39)	ISH: Virus in intesrtitial cells and macrophages	
Tavazzi et al. (2020)	1 (M; 69 years)	N.A. (biopsy)	Interstitial and endocardial inflammation, focal myofibrillar lysis, macrophages infiltration	CD68 (positive)	N.A.	TEM: Membrane damage and cytoplasmic vacuoles, single or small groups of viral particles with the morphology of coronaviruses	

N.A., not available; ATTR, amyloid transthyretin; ISH, *in situ* hybridization; TEM, transmission electron microscopy.

### Liver Findings

The gross examination of the liver showed signs of steatosis (Lax et al., 2020; Menter et al., 2020) as the most frequent finding, while in some cases, signs of shock necrosis (Menter et al., 2020) were observed. In one case, a macroscopic infarction was detected (Hanley et al., 2020b). The most frequent microscopic findings included steatosis (Wang X.-X. et al., 2020; Wang et al., 2020a; Lacy et al., 2020; Lax et al., 2020; Nunes Duarte-Neto et al., 2020; Oprinca and Muja, 2020; Prilutskiy et al., 2020; Remmelink et al., 2020; Sonzogni et al., 2020), chronic congestion (Lacy et al., 2020; Lax et al., 2020; Nunes Duarte-Neto et al., 2020; Prilutskiy et al., 2020), lymphocytic infiltrates especially in the portal/periportal tract (Wang et al., 2020a; Lax et al., 2020; Remmelink et al., 2020; Schaller et al., 2020; Sonzogni et al., 2020; Varga et al., 2020), hepatocyte necrosis (Wang X.-X. et al., 2020; Lax et al., 2020; Nunes Duarte-Neto et al., 2020; Sonzogni et al., 2020; Varga et al., 2020), hyperplasia, and hypertrophy of the Kupffer cells (Lax et al., 2020; Nunes Duarte-Neto et al., 2020; Prilutskiy et al., 2020). Less reported findings were central lobular pallor (Lacy et al., 2020), cholestasis, and ductular proliferation (Lax et al., 2020), focal lobular inflammation with predominant lymphocytes (Wang et al., 2020a; Sonzogni et al., 2020). Rapkiewicz et al. (2020) observed platelet-fibrin microthrombi in hepatic sinusoids and larger platelet aggregates in the portal veins. Sonzogni et al. (2020) reported variable degrees of portal vein endotheliitis, diffuse alterations of intrahepatic vascular structures (portal branches and sinusoids) and variable degrees of partial/complete luminal thrombosis. Wang et al. (2020a) studied the hepatocyte ultrastructural morphology in two different liver samples, revealing the presence of typical coronavirus particles in the cytoplasm mostly without membrane-bound vesicles. The schematic summary of the data about the liver is reported in Table 3.

**TABLE 3:** Main data on liver findings associated with COVID-19 deaths reported in analyzed articles.

Author(s)	Sample	Gross examination	Microscopic finding(s)	Immunoistochemistry	Post-mortem molecular test	Other(s)
Lacy et al. (2020)	1 (F, 58 years)	Unrelevant	Mild steatosis, central lobular pallor and congestion	N.A.	N.A.	N.A.
Menter et al. (2020)	21 (4 F; 17 M; 53–96 years))	N.A.	Steatosis (7/17), shock necrosis (5/17), alcoholic steatohepatitis (ASH)/Non-ASH (3/17)	N.A.	N.A.	N.A.
Lax et al. (2020)	11 (8 m; 3 F; 66–91 years)	Steatosis (11/11)	Steatosis (11/11), chronic congestion (8/11), hepatocyte necrosis (7/11), kupffer cell proliferation (10/10), cholestasis (8/11), fibrosis (6/11), lymphocytic infiltrate (8/11), ductular proliferation (8/11)	N.A.	N.A.	N.A.
Oprinca and Muja (2020)	2 (1 F 79; 1 M 70 years)	Hepatomegaly	Vascular congestion, macro-vesicular steatosis, mild to moderate lymphocytic infiltrate	N.A.	N.A.	N.A.
Remmelink et al. (2020)	17 (12 M, 5 F; 62–77 years)	Hepatomegaly (5/17)	Congestive hepatopathy (7/17), liver cirrhosis (2/17), hepatic steatosis (10/17)	N.A.	RT-PCR (positive 11/16)	N.A.
Nunes Duarte-Neto et al. (2020)	10 (5 F; 5 M; 33–83 years)	N.A. (biopsy)	Steatosis (6/10), portal tract inflammatory infiltrate (9/10), centrilobular congestion (10/10), ischemic necrosis (3/10), kupffer cell hypertrophy (5/10) and hemophagocytosis (3/10)	N.A.	N.A.	N.A.
Hanley et al. (2020a)	10 (7 M, 3 F; 73 years median age) thereof 1 MIA biopsy	Hepatomegaly (3/9), liver infarction (1/9)	Cirrhosis or bridging hepatic fibrosis (3/9)	N.A.	RT-PCR (positive 3/5)	N.A.
Schaller et al. 2020	10 (3 F, 7 M; 64–90 years)	N.A.	Periportal lymphoplasma cellular infiltration, signs of fibrosis	N.A.	N.A.	N.A.
Wang et al. (2020a)	2 (1 F, 79 years; 1 M, 50 years)	N.A. (biopsy)	Apoptotic hepatocytes, prominent binuclear or multinuclear syncytial hepatocytes, microvescicular and macrovesicular steatosis, focal lobular inflammation with infiltration of predominant lymphocytes and few neutrophils, mild inflammation in the portal tracts with lymphocytic infiltrate	CD68, CD4, CD8, Ki67 (positive)	N.A.	TEM: Coronavirus particles in hepatocyte cytoplasm, most viral particles without membrane-bound vesicles. TUNEL: Positive cells in nuclei
Prilutskiy et al. (2020)	4 (1 F; 3 M; 64–91 years)	Unrelevant	Centrolobular congestion and steatosis	Pax-5, CD3 (negative); CD163 (positive)	N.A.	N.A.
Wang et al. (2020b)	1 (F, 75 years)	N.A. (biopsy)	Coagulative necrosis, microvesicular steatosis, apoptosis, canalicular cholestasis	N.A.	N.A.	ISH (negative)
Rapkiewicz et al. (2020)	7 (4 F, 3 M; 44.65 years)	Mild macrovesicular steatosis (7/7)	Mild, macrovesicular steatosis (7/7), cirrhosis (1/7), platelet-fibrin microthrombi in hepatic sinusoids (6/7) with ischemic type hepatic necrosis (2/7), platelet aggregates in the portal veins (2/7)	N.A.	N.A.	N.A.
Varga et al. (2020)	3 (M, 71 years; F, 58 years; M, 69 years)	N.A.	Lymphocytic endotheliitis and cell necrosis (case 2)	N.A.	N.A.	N.A.
Sonzogni et al. (2020)	48 (35 M, 13 F; 32–86 years)	N.A.	Portal vein parietal fibrosis (29/48); herniated portal vein in periportal. Parenchyma (36/48); periportal abnormal vessels (48/48); fibrosis (37/48); lobular inflammation (24/48); portal inflammation (32/48); vascular thrombosis (porta 25/48; sinusoidal 13/48); parenchymal confluent necrosis (18/48); steatosis (26/48)	CD3, CD4, CD20 (positive); CD34 (positive); factor VIII, SMA (positive); C4d (negative)	N.A.	ISH (15/22 in blood cloths or endothelial cells)

N.A., not available; ISH, *in situ* hybridization; TEM, transmission electron microscopy; TUNEL, terminal deoxynucleotidyl transferase dUTP nick end labeling; SMA, actin smooth muscle.

### Kidney Findings

The gross examination of kidneys did not reveal any particular finding. The most frequent and relevant microscopic evidence included acute tubular damage (Hanley et al., 2020b; Wang X.-X. et al., 2020; Kudose et al., 2020; Lax et al., 2020; Menter et al., 2020; Oprinca and Muja, 2020; Nunes; Oprinca and Muja, 2020; Santoriello et al., 2020; Sekulic et al., 2020; Su et al., 2020; Yan et al., 2020) and fibrin microthrombi in glomeruli (Hanley et al., 2020b; Nunes Duarte-Neto et al., 2020; Oprinca and Muja, 2020; Rapkiewicz et al., 2020; Santoriello et al., 2020; Su et al., 2020). Yan et al. (2020) described a focal acute tubular injury with flattened epithelium and lumens containing sloughed epithelial lining cells, granular casts, Tamm-Horsfall protein, and intraluminal accumulation of cellular debris in focal areas. Other less frequent changes were disseminated intravascular coagulation (Menter et al., 2020), hemosiderin in renal tubules (Remmelink et al., 2020), chronic interstitial inflammation with sporadic prominent perivascular lymphocytic inflammation (Bradley et al., 2020), hypertensive and diabetic nephropathy (Menter et al., 2020), and unspecific nephrosclerosis (Lax et al., 2020).

Su et al. (2020) evaluated six kidney samples using immunofluorescent stain with positive results for SARS-CoV-2 NP in 50% of the cases and, using TEM, observed coronavirus-like particles in 7 out of 9 cases together with dense deposit and subendothelial expansion. Other researchers also performed TEM, which revealed prominent activation of podocytes with multiple cytoplasmic vesicles containing virus-like particles, also detected in endothelial cells and proximal tubular epithelial cells (Bradley et al., 2020; Menter et al., 2020). The virions were also detected in proximal convoluted tubules (Rapkiewicz et al., 2020).

All the main kidney findings are reported in Table 4.

**TABLE 4:** Kidney evidences described in COVID-19 death.

Author(s)	Sample	Gross examination	Microscopic finding(s)	Immunoistochemistry	Post-mortem molecular test	Other(s)
Bradley et al. (2020)	14 (7 M, 7 F; 42–84 years)	N.A.	Chronic interstitial inflammation, more prominent perivascular lymphocytic inflammation	SARS-CoV-2 (2/14 in renal tubular epithelial cells)	RT-PCR (positive)	TEM: Viral particles in endothelial cells (1/2), proximal tubular epithelial cells (1/2)
Sekulic et al. (2020)	2 (2 M; 54 and 81 years)	N.A.	ATI	N.A.	RT-PCR (negative)	N.A.
Lacy et al. (2020)	1 (F, 58 years)	Finely granular, focal cortical scars	Arteriolosclerosis, mesangial sclerosis, hypercellularity, focal global glomerulosclerosis	N.A.	N.A.	N.A.
Yan et al. (2020)	1 (F, 44 years)	Unrelevant	Focal ATI with tubules flattened epithelium, lumens containing sloughed epithelial lining cells, granular casts, tamm-horsfall protein and intraluminal accumulation of cellular debris in focal areas	N.A.	N.A.	N.A.
Menter et al. (2020)	21 (4 F; 17 M; 53–96 years)	Unrelevant	ATI (14/15), DIC (3/17), hypertensive nephropathy (2/17), Diabetic nephropathy (2/17)	N.A.	RT-qPCR (variable)	TEM: Prominent activation of podocytes, endothelial cells and proximal tubular epithelial cells; vesicles in podocytes cytoplasm with virus-like particles (2/2)
Lax et al. (2020)	11 (8 M; 3 F; 66–91 years)	N.A.	Nodular glomerulosclerosis (4/11), benign nephrosclerosis (10/11), ATI with necrosis (11/11), chronic interstitial nephritis (2/11); cortical fibrosis (1/11)	N.A.	N.A.	N.A.
Oprinca and Muja (2020)	3 (1 F 79 years; 1 F 27 years; 1 M 70 years)	Medullary congestion (2/3)	ATI, focal microthrombi in glomeruli (2/2)	N.A.	N.A.	N.A.
Remmelink et al. (2020)	17 (12 M, 5 F; 62–77 years)	Enlarged with a pale cortex and petechial aspect	Hemosiderin renal tubules (9/17), pigmented renal casts (12/17)	N.A.	RT-PCR (positive 14/16)	N.A.
Nunes Duarte-Neto et al. (2020)	10 (5 F; 5 M; 33–83 years)	N.A. (biopsy on 8/10)	Glomeruli shrinkage (8/8), fibrin thrombi (6/8), focal and mesangial matrix expanding (8/8), focal glomerular sclerosis (7/8), ATI (8/8), hyaline tubular casts (6/8), pigmented tubular casts (1/8), arteriolosclerosis (8/8)	N.A.	N.A.	N.A.
Hanley et al. (2020a)	10 (7 M, 3 F; 73 years median age)	Unrelevant	ATI, glomerular microaneurysm and thrombi (1/9), rare thrombi in interlobular arteries	N.A.	RT-PCR (positive 3/4)	N.A.
Rapkiewicz et al. (2020)	7 (4 F, 3 M; 44.65 years)	N.A.	Microthrombi in scattered peritubular capillaries and venules, ATI with necrosis, cellular casts, some pigmented red blood cell casts (7/7); thrombotic microangiopathy in glomeruli (1/7)	C4d (negative 2/2)	N.A.	TEM: Virions in proximal convoluted tubules, rare podocyte virions
Kudose et al. (2020)	17 (5 F, 12 M; 22–72 years)	N.A. (biopsy)	ATI, collapsing glomerulopathy, membranous glomerulopathy	Sars-CoV-2 sP and NP (negative)	N.A.	TEM: Tubular reticular inclusion
Su et al. (2020)	26 (7 F; 19 M; 39–87 years)	N.A.	ATI (26/26), multiple foci of bacteria (2/26), pigmented casts (3/26), arteriosclerosis (26/26), glomerular segmental fibrin thrombus with severe endothelial injury (3/26), focal segmental glomerulosclerosis (2/26)	CD3, CD4, CD8, CD20, CD21, CD31, CD61, CD68 (positive, 9/9), ACE2 (positive in proximal tubules, 3/5). IF: SARS-CoV-2 NP (3/6)	N.A.	TEM: Coronavirus-like particles (7/9), dense deposit (2/9), subendothelial expansion (5/9)
Santoriello et al. (2020)	42 (29 M, 13 F; 38–97 years)	N.A.	ATI (19/32), focal fibrin thrombi in glomeruli or blood vessel (6/42)	N.A.	N.A.	TEM: Tubules degenerative changes with attenuation and loss of brush border, dilation of endoplasmic reticulum and intraluminal cellular debris. ISH: Negative

N.A., not available; ATI, acute tubular injury; Sars-CoV-2 NP, Sars-CoV-2 nucleo-capsid protein; Sars-CoV-2 sP, Sars-CoV-2 spike protein; ACE2, angiotensin-converting enzyme 2; IF, immunofluorescence; TEM, transmission electron microscopy.

### Other Organs

Data on brain involvement in COVID-19 are controversial. In particular, in a study conducted on six autopsy cases, von Weyhern et al. (2020) observed massive intracranial hemorrhage and diffuse petechial hemorrhages along with microscopic findings of localized perivascular and interstitial encephalitis, neuronal cell loss, and axon degeneration of multiple neuronal areas. Remmelink et al. (2020) described cerebral focal necrosis and cerebral hemorrhage. Similarly, another study (Reichard et al., 2020) on a single case described destructive hemorrhagic white matter lesions, focal microscopic necrosis, perivascular cellular infiltrates, and axonal injury, then confirmed by the immunohistochemical positivity to different markers such as CD68, CD3, CD20, GFAP (glial fibrillary acidic protein), APP (amyloid precursor protein) and PLP (myelin proteolipid protein). Conklin et al. (2020) reported microscopic ischemic lesions associated with widespread microvascular injuries as perivascular and parenchymal petechial hemorrhages. The ischemic damage was also found in another report by the BAPP (β amyloid precursor protein) immunohistochemical stain and T-cell infiltration around blood vessels and capillaries (Hanley et al., 2020b). Similar findings were described by Kantonen et al. (2020), reporting enlarged perivascular spaces, microhemorrhages, scattered T-lymphocytes, and minor intravascular fibrinoid deposits in some cerebral and subarachnoidal vessels. Moreover, in the nine autopsy cases examined by Nunes Duarte-Neto et al. (2020), reactive gliosis, neuronal satellitosis, small vessel disease, and perivascular hemorrhages were reported. On the contrary, Solomon et al. (2020) described only acute hypoxic-ischemic damage in the absence of microscopic specific elements; however, the same research group highlighted negative SARS-Cov-2 immunohistochemistry and positive molecular diagnosis by RT-PCR in few samples of the medulla, olfactory nerves, and frontal lobe.

Some reports described macroscopic and histological evidence of the spleen. Oprinca and Muja (2020) reported histologically marked congestion and white pulp atrophy associated with the absence of lymphoid follicles. Prilutskiy et al. (2020) observed an enlarged, soft, and friable organ just in one of the four analyzed cases. Microscopically, they described white pulp depletion with red pulp hemorrhage or infarction and histiocytic hyperplasia with hemosiderin-laden macrophages, suggestive of a prior red blood cells phagocytosis, or hyperplastic white pulp with red pulp congestion but lacking hemophagocytosis. White pulp depletion and red pulp hemorrhage were reported also by Rapkiewicz et al. (2020). Even Nunes Duarte-Neto et al. (2020) studied the spleen in five cases reporting lymphoid hypoplasia, red pulp hemorrhages, and splenitis.

Additionally, they described follicular arterioles endothelial changes, vasculitis, and arterial thrombus. Likewise, an acute splenitis was observed by Menter et al. (2020) in six out of 21 cases, while Lax et al. (2020) found lymphocyte depletion affecting both the spleen and lymph nodes. Furthermore, in two cases necrotizing granulomata was reported (Sekulic et al., 2020) in the spleen.

Lymph nodes and bone marrow histological changes have been observed in two of the above-mentioned studies. Indeed, one of these (Prilutskiy et al., 2020) described enlarged mediastinal and pulmonary lymph nodes that showed a hemophagocytic histiocytes CD163+, while the other (Menter et al., 2020) reported lymph nodes congestion and increased presence of plasmablasts. As for the bone marrow, both research groups reported left-shifted myeloid hyperplasia; in addition, Prilutskiy et al., 2020) also observed histiocytic cells CD163+. Wang X.-X. et al. (2020) reported data on lymphoid tissue describing primary lymphoid follicle, scattered T lymphocytes, and focal necrosis. The virions detection in bone marrow was reported only in one study using TEM, which detected megakaryocytes (Rapkiewicz et al., 2020).

Other interesting evidence was provided by Varga et al. (2020), who described mesenteric ischemia and small bowel sub-mucosal vessels endotheliitis. One case of ischemic enteritis was also reported (Remmelink et al., 2020). Ischemic bowel changes were observed by Skok et al. (2020), namely atrophic cripts, cryptitis, ulceration, and hemorrhage. Some cases of pancreatitis were also detected (Hanley et al., 2020b; Lax et al., 2020).

Adrenal gland findings were reported by Iuga et al. (2020), who described small vessels with acute fibrinoid necrosis, subendothelial vacuolization, and apoptotic debris. In a further study (Lax et al., 2020), adrenal cortical hyperplasia was described. Interestingly, Hanley et al. (2020b) described patchy areas of infarct-type adrenocortical necrosis and organizing microthrombi in adrenal vessels.

Furthermore, Yang et al. (2020) studied the tests in 12 cases using post-mortem biopsy, detecting Sertoli cells swelling, reduced Leydig cells, mild lymphocytic inflammation, detachment from tubular basement membranes and lumen intratubular cell mass loss and sloughing; in the same study, the immunohistochemical positivity to different markers such as CD3, CD20, CD68, CD138, and ACE-2 was observed, but the RT-PCR confirmed the presence of the virus only in a biopsy sample. Nevertheless, Nunes Duarte-Neto et al. (2020), in two cases out of two, observed an orchitis condition.

Finally, the involvement of the skin was included in the study performed by Magro et al. (2020). Five cases with purpuric lesions were described, microscopically characterized by thrombogenic vasculopathy, epidermis, and adnexal structures necrosis, interstitial and perivascular neutrophilia with prominent leukocytoclasia or perivascular lymphocytic infiltrate in the superficial dermis with small thrombi within rare venules of the deep dermis. The same study highlighted the immunohistochemical positivity to different markers like C4d, C3d, C5b-9, MASP2, and SARS-CoV-2 spike and envelope proteins. Purpuric lesions, superficial perivascular mononuclear infiltrate, and endothelial changes were also described by another work in which interesting findings in the skeletal muscle were evaluated, consisting of myositis and necrotic fibers (Nunes Duarte-Neto et al., 2020).

The findings about the mentioned organs are summarized in Table 5.

**TABLE 5:** Summary of the main findings on other organs/tissues described in Sars-CoV-2 related death.

Author(s)	Sample	Organ(s)	Microscopic finding(s)	Immunoistochemistry	Post-mortem molecular test	Other(s)	
Sekulic et al. (2020)	2 (2 M; 54, 81 years)	Spleen: Enlarged, congested (1/2)	Necrotizing granulomata (1/2)	N.A.	RT-PCR (positive)	N.A.	
Magro et al. (2020)	5 (2 F, 3 M; 32–73 years)	SKIN: Purpuric lesions (3/5)	Thrombogenic vasculopathy, epidermis and adnexal structures necrosis, interstitial and perivascular neutrophilia with prominent leukocytoclasia (1/3); superficial vascular ectasia and an occlusive arterial thrombus in the deeper reticular dermis (1/3); perivascular lymphocytic infiltrate in superficial dermis with small thrombi within rare venules of the deep dermis (1/3)	C4d, C5b-9 (positive 3/3); SARS-cov-2 sP and eP (positive 2/3)	N.A.	N.A.	
Menter et al. (2020)	21 (4 F; 17 M; 53–96 years)	LYMPH NODE - SPLEEN - BONE MARROW	LYMPH NODE: Plasmablasts increase (5/9), congestion (6/9); SPLEEN: Acute splenitis (6/21); BONE MARROW: Reactive left shift of myelopoiesis (3/5)	CD3, CD4, CD8, CD20, CD68, multiple myeloma 1 (positive)	N.A.	N.A.	
Lax et al. (2020)	11 (8 m; 3 F; 66–91 years)	PANCREAS - ADRENAL GLAND - SPLEEN - LYMPH NODE	Pancreas: Focal pancreatitis (5/11); ADRENAL GLAND: Cortical hyperplasia (6/8); SLEEN: Lymphocyte depletion (10/11); LYMPH NODE: Lymphocyte depletion (11/11)	N.A.	N.A.	N.A	
Nunes Duarte-Neto et al. (2020)	10 (5 F; 5 M; 33–83 years)	BRAIN - SPLEEN - SKIN - SKELETAL MUSCLE -testis	BRAIN (n = 9): Reactive gliosis (8/9), neuronal satellitosis (5/9), small vessels disease (3/9), perivascular hemorrhages (1/9); SPLEEN (n = 5): Lymphoid hypoplasia (5/5), red pulp hemorrhages (3/5), splenitis (2/5), extramedullary hematopoiesis (5/5), endothelial changes of follicular arterioles (4/5), vasculitis and arterial thrombus (1/5); SKIN: Superficial perivascular mononuclear infiltrate (8/10), purpura (1/10), endothelial changes (3/10); SKELETAL MUSCLES: Myositis (6/10), necrotic fibers (8/10); TESTIS (n = 2): Orchitis (2/2)	N.A.	N.A.	N.A.	
von Weyhern et al. (2020)	6 (2 F, 4 M; 58–82 years)	Brain: Massive intracranial hemorrhage (2/6), diffuse petechial hemorrhage	Lymphocytic pan-encephalitis and meningitis; localized perivascular and interstitial encephalitis, neuronal cell loss, axon degeneration in the dorsal motor nuclei of the vagus nerve, CN V, nucleus tractus solitarii, dorsal raphe nuclei, and fasciculus longitudinalis medialis	N.A.	N.A.	TEM: Unrelevant findings	
Prilutskiy et al. (2020)	4 (1 F; 3 M; 64–91 years)	Spleen: Enlarged, soft and friable (1/4); LYMPH NODES: Enlarged	Spleen: Red pulp hemorrhage with admixed phagocytic histiocytes, focal hemophagocytosis and white pulp depletion (1/4); white pulp depletion with red pulp infarction, histiocytic hyperplasia, and numerous hemosiderin-laden macrophages (1/4); hyperplastic white pulp with red pulp congestion (2/4). BONE marrow: Trilineage hematopoiesis with left-shifted myeloid hyperplasia (2/2). LYMPH nodes: Clusters of hemophagocytic histiocytes, lymphophagocytosis predominantly	CD163 (positive in bone marrow and lymph nodes)	N.A.	N.A.	
Varga et al. (2020)	3 (M, 71 years; F, 58 years) + M, 69 years (small intestine resection)	SMALL INTESTINE	Mesenteric ischemia and submucosal vessels endotheliitis (1/3); endothelialitis (1/3)	N.A.	N.A.	N.A.	
Reichard et al. (2020)	1 (M; 71 years)	Brain: Swelling, hemorrhagic lesions	Destructive hemorrhagic white matter lesion with white matter pallor adjacent and peripheral macrophages, axonal injury, focal microscopic necrosis, perivascular cellular infiltrates	CD68, CD3, CD20, glial fibrillary acidic protein (GFAP), amyloid precursor protein (APP), myelin proteolipid protein (PLP) (positive)	N.A.	N.A.	
Conklin et al. (2020)	1 (N.A.)	BRAIN	Widespread microvascular injury of white matter with perivascular and parenchymal petechial hemorrhages and microscopic ischemic lesions	N.A.	N.A.	N.A.	
Solomon et al. (2020)	18 (4 F; 14 M; 53–75 years)	Brain: Unrelevant	Acute hypoxic ischemic damage (14/14)	SARS-CoV-2 (negative)	RT-PCR (positive in medulla, frontal lobe and olfactory nerves 16/18)	N.A.	
Iuga et al. (2020)	5 (4 F, 1 M; 59–90 years)	ADRENAL GLAND	Small vessels with acute fibrinoid necrosis, subendothelial vacuolization and apoptotic debris were present	N.A.	N.A.	N.A.	
Wang et al. (2020b)	1 (M; 65 years)	LYMPH NODE	Primary lymphoid follicles, scattered T lymphocytes, focal necrosis, nuclear fragmentation	CD3, CD4, CD8 (positive)	RT-PCR (negative)	N.A.	
Oprinca and Muja, 2020)	2 (1 F 79; 1 M 70 years)	Spleen: Amyloid deposits on the surface (1/3) - LYMPH NODES: Pulmonary hilar and mediastinal lymphadenopathies (1/3)	Marked congestion white pulp atrophy absence of lymphoid follicles (2/3)	N.A.	N.A.	-	
Hanley et al. (2020a)	10 (7 M, 3 F; 73 years median age) thereof 1 MIA biopsy	SPLEEN - BONE MARROW - LYMPH NODE - ADRENAL GLAND - BRAIN	Spleen: Increased phagocytosis of other cells in red pulp sinusoidal macrophages (4/7), depletion of periarteriolar T-cell in white pulp (7/7),CD8-t cells reduced in red pulp (7/7), increasing of plasma cells, sinusoidal histiocytes phagocytosis of cells (7/7). BONE marrow: Hemophagocytosis (4/7), trilineage hyperplasia with plasma cells and histiocytes increasing (7/7). LYMPH nodes: Paracortical areas depletion (7/7). Adrenals: Patchy areas of infarct-type adrenocortical necrosis (3/9), vessels organizing microthrombi (1/9). Brain: Moderate to intense microglial activation (5/5), mild T-cell infiltration was noted around blood vessels and capillaries (5/5), ischemic neuronal changes (5/5)	CD3, CD4, CD20, CD34, CD56, CD68-PGM1, CD61 (positive). Brain: BAPP (positive)	RT-PCR (bone marrow: Positive 1/3. Brain: Positive 4/5. Spleen: Positive 2/3)	N.A.	
Remmelink et al. (2020)	17 (12 M, 5 F; 62–77 years)	Gut: Ischemic enteritis (1/17). Brain: Subdural hematoma (1/17) and another a cerebral hemorrhage (1/17)	BONE marrow: Hyperplasia (14/17). Brain: Cerebral focal necrosis (3/11), cerebral hemorrhage (2/11), cerebral edema (5/11), cerebral spongiosis (10/11). Bowel: Inschemic enteritis (1/11)	N.A.	RT-PCR (spleen: Positive 11/16. Bowel: Positive 14/17. Brain: Positive 9/11)	N.A.	
Rapkiewicz et al. (2020)	7 (4 F, 3 M; 44.65 years)	Spleen: Congestion (1/7) - BONE MARROW - LYMPH NODE	Spleen: White pulp depletion and red pulp congestion (n.a.). BONE marrow: Hypercellular with increased megakaryocytes (n.a.). LIMPH nodes: Dilated sinuses with marked sinus histiocytosis with focal erythrophagocytosis, numerous platelets, and megakaryocytes (5/5)	LIMPH nodes: CD61 (positive)	N.A.	TEM: Rare virions in bone marrow megakaryocytes	
Kantonen et al. (2020)	4 (3 M, 1 F; 63–90 years)	Brain: Swelling, depigmentation of the substantia nigra and locus coeruleus, discoloration of the watershed areas, and a few lacunae in inferior putamen (1/4)	Brain: Enlarged perivascular spaces with hemosiderophages, acute microhemorrhages, scattered T lymphocytes with very few B lymphocytes, fibrinoid deposits in cerebral and subarachnoidal vessels (1/4)	SARS- CoV-2 NP (negative)	RT-PCR (negative)	N.A.	
Skok et al. (2020)	19 (7 M, 12 F; 66–93 years)	BOWEL	Bowel: Trophic crypts, cryptitis, ulceration, and hemorrhage (6/19)	anti-SARS- NP (n.a.)	RT-PCR (positive 5/11)	N.A.	
Yang et al. (2020)	12 (M; 39–87 years)	TESTE (biopsy)	Sertoli cells swelling, vacuolation and cytoplasmic rarefaction, detachment from tubular basement membranes, lumen intratubular cell mass loss and sloughing, reduced leydig cells, mild lymphocytic inflammation	CD3, CD20, CD68, CD138, ACE2 (positive)	RT-PCR (positive 1/12)	TEM: Unrelevant findings (3/12)	

N.A., not available; Sars-CoV-2 NP, Sars-CoV-2 nucleo-capsid protein; Sars-CoV-2 sP, Sars-CoV-2 spike protein; Sars-CoV-2 eP, Sars-CoV-2 envelope protein; GFAP, glial fibrillary acidic protein; APP, amyloid precursor protein; PLP, myelin proteolipid protein; BAPP, *β* amyloid precursor protein; ACE2, angiotensin-converting enzyme 2; TEM, transmission electron microscopy.

## Discussion

In this work, we reported the main findings related to COVID-19 tissues. The authors reviewed studies describing the macroscopic and microscopic (histological and immunohistochemical) data and the ultrastructural and molecular evidence observed in different organs by both biopsies and autopsies. Only the histological samples and, in general, the autopsy findings enable us to ascertain the exact cause of death, especially in suspect or probable cases ofSARS-CoV-2 infection; moreover, the post-mortem evidence provides fundamental data which are useful to better understand the pathophysiology of this infection; this can help clinicians identify the most appropriate and effective treatment to reduce mortality.

Although documented by clinical and imaging data of patients affected by this infection (Lake, 2020), the revision revealed that the lung is the most affected organ, showing injuries similar to those observed in diseases related to other coronaviruses, the Severe Acute Respiratory Syndrome (SARS) and the Middle East Respiratory Syndrome (MERS) (Ding et al., 2003; Ng et al., 2016), even if the alterations related to COVID-19 are more extensive and severe, and collectively constitute a distinctive feature (Buja et al., 2020).

In critical cases, the acute COVID-19 respiratory picture revealed a severe acute respiratory distress syndrome (ARDS) associated with the histological detection of DAD in different stages, mainly in exudative and proliferative phases. In particular, this condition was characterized by hyaline membranes, intra-alveolar and/or septal and/or interstitial edema, intra-alveolar fibrinous exudate and inflammatory cells infiltrates, type 2 pneumocyte hyperplasia/activation, and squamous metaplasia (Okudela et al., 2020; Wichmann et al., 2020; Heinrich et al., 2020; Bradley et al., 2020; Aguiar et al., 2020; Sekulic et al., 2020; Cipolloni et al., 2020; Edler et al., 2020; Suess and Hausmann, 2020; Navarro Conde et al., 2020; Carsana et al., 2020; Youd and Moore, 2020; Lacy et al., 2020; Yan et al., 2020; Konopka et al., 2020b; Menter et al., 2020; Fox et al., 2020; Buja et al., 2020; Oprinca and Muja, 2020; Fitzek et al., 2020; Remmelink et al., 2020; Schaefer et al., 2020; Nunes Duarte-Neto et al., 2020; Grimes et al., 2020; Hanley et al., 2020b; Wang C. et al., 2020; Aiolfi et al., 2020; Martines et al., 2020; Xu et al., 2020; Tian et al., 2020a; Tian et al., 2020b; Karami et al., 2020; Schaller et al., 2020; Cai et al., 2020; von Weyhern et al., 2020; Shao et al., 2020; Dolhnikoff et al., 2020; Konopka et al., 2020a; Ackermann et al., 2020; Wang et al., 2020a; Prilutskiy et al., 2020; Barton et al., 2020; Zhang et al., 2020; Wang X.-X. et al., 2020; Rapkiewicz et al., 2020; Kantonen et al., 2020).

Immunohistochemical analysis was fundamental to better define the immune cells infiltrating the lung tissue (Heinrich et al., 2020; Aguiar et al., 2020; Cipolloni et al., 2020; Suess and Hausmann, 2020; Navarro Conde et al., 2020; Carsana et al., 2020; Menter et al., 2020; Fox et al., 2020; Buja et al., 2020; Oprinca and Muja, 2020; Nunes Duarte-Neto et al., 2020; Grimes et al., 2020; Hanley et al., 2020b; Aiolfi et al., 2020; von Weyhern et al., 2020; Ackermann et al., 2020; Barton et al., 2020; Wang X.-X. et al., 2020; Rapkiewicz et al., 2020; Varga et al., 2020), it showed all aspects of innate immune response and T and B cells immunity once the virus has entered the tissue cells. In fact, it was reported that, in COVID-19, white cells immunity acts as in a classical respiratory virus-like infection (Jansen et al., 2019; Azkur et al., 2020): recognition of the whole virus and viral particles by professional antigen-presenting cells, which are mainly dendritic cells and macrophages, that present viral peptides to CD4^+^ T cells; activation of CD8^+^ cytotoxic T cells to lyse the virus-infected cells; activation of B cells that can directly recognize the viruses and also interact with CD4^+^ T cells. Additionally, other two investigated markers were ACE-2 and TTF-1 on which some interesting consideration can be carried out. ACE-2 was recognized as a receptor for host cell entry of SARS-CoV-2, being positive in alveolar epithelial, endothelial cells, alveolar macrophages, and lymphocytes in lung tissue samples (Ackermann et al., 2020; Cipolloni et al., 2020; Grimes et al., 2020). It was also reported that both the tissue activity and membrane expression of ACE-2 increase the susceptibility to COVID-19. On the contrary, several studies indicated that downregulation of ACE-2 is associated with the aggravation of inflammatory events due to both anti-inflammatory and anti-fibrotic actions of ACE-2 activators and the modifications in renin-angiotensin-aldosterone system function (Behl et al., 2020). TTF-1 (thyroid transcription factor-1) was used to evaluate the pneumocytes, resulting positive in those hyperplastic and dysmorphic (Aiolfi et al., 2020; Buja et al., 2020; Carsana et al., 2020; Fox et al., 2020; Menter et al., 2020; Navarro Conde et al., 2020; Nunes Duarte-Neto et al., 2020; Schaefer et al., 2020; Shao et al., 2020; Suess and Hausmann, 2020). Notably, this marker was detected in dysmorphic cells showing features of syncytia, characterized by the presence of multi-nucleation and an ample cytoplasm surrounded by a single plasmalemma; these syncytial elements of pneumocytic origin are considered characteristic of COVID-19 (Bussani et al., 2020).

The pulmonary thrombotic microangiopathy is another observed microscopic pattern, together with DAD, in several cases resulting often in fibrin-platelet thrombi in alveolar capillaries and/or in small vessels (Konopka et al., 2020a; Barton et al., 2020; Konopka et al., 2020b; Bradley et al., 2020; Buja et al., 2020; Cai et al., 2020; Carsana et al., 2020; Dolhnikoff et al., 2020; Fox et al., 2020; Grimes et al., 2020; Kantonen et al., 2020; Menter et al., 2020; Navarro Conde et al., 2020; Nunes Duarte-Neto et al., 2020; Oprinca and Muja, 2020; Rapkiewicz et al., 2020; Remmelink et al., 2020; Santana et al., 2020; Schaefer et al., 2020; Shao et al., 2020; Suess and Hausmann, 2020; Wichmann et al., 2020) associated with endothelial damage showing different features (i.e. thrombotic necrotizing capillary injury, perivascular inflammation/endothelialitis) (Ackermann et al., 2020; Cai et al., 2020; Dolhnikoff et al., 2020; Edler et al., 2020; Wang et al., 2020a; Magro et al., 2020; Menter et al., 2020; Remmelink et al., 2020; Varga et al., 2020). In particular, Achermann et al. (Ackermann et al., 2020) reported distinctive angiocentric features in some cases of COVID-19 represented by severe endothelial injury associated with intracellular SARS-CoV-2 virus and endothelial cell membranes destruction, widespread vascular thrombosis with microangiopathy and alveolar capillaries occlusion, a significant new vessel growth by intussusceptive angiogenesis. The latter morphological/pathological aspect suggests a severe degree of endothelialitis and thrombosis in the lung. Very interesting evidence was described by Magro et al. (2020) because the authors analyzed pulmonary abnormalities largely restricted to the alveolar capillary with immunohistochemical detection of complement components and MAC in the lung septal microvasculature, this suggested an “atypical ARDS” in which a catastrophic microvascular injury syndrome occurs activating the complement pathway. Moreover, this would be supported by C5b-9 involvement in pathologies as antiphospholipid antibody syndrome and purpura fulminans (Perera and Murphy-Lavoie, 2020; Ruffatti et al., 2020). MAC contributes to endothelial activation, resulting in cell adhesion molecules and releasing chemotactic and activating factors related to migration and activation of white cells (Kilgore et al., 1995; Mondello et al., 2017; Mondello et al., 2018; Mondello et al., 2020). The role of the complement system in lung injuries due to SARS-CoV-2 was also supported by the immunohistochemical detection of MASP-2 (mannan-binding lectin-associated serine protease-2) (Magro et al., 2020), an effector enzyme of the lectin pathways capable of binding the SARS-CoV-2 nucleocapsid protein, resulting in complement activation and endothelial/tissue injury (Rambaldi et al., 2020).

Some researchers completed the microscopic data evaluating the presence of the virus in the analyzed lung tissue. SARS-CoV-2 was observed in pneumocytes, alveolar macrophages, intralveolar septa, and septal capillary by immunohistochemistry performed using antibodies for nucleocapsid protein or spike and envelope proteins (Bradley et al., 2020; Magro et al., 2020; Martines et al., 2020; Rapkiewicz et al., 2020; Remmelink et al., 2020; Schaefer et al., 2020; Zhang et al., 2020). These virus localizations were also confirmed by electron microscopy (Bradley et al., 2020; Carsana et al., 2020; Fox et al., 2020; Martines et al., 2020; Rapkiewicz et al., 2020; Yan et al., 2020) as viral particles were found either outside or inside the cells in aggregates confined within vesicles (Bradley et al., 2020).

Current collected evidence integrates and completes the clinical studies on understanding the pathological mechanism underlying the SARS-CoV-2 infection in which both the innate and the acquired immune system (i.e. tissue barriers, innate and adaptive cells, and mediators) are involved (Azkur et al., 2020). Currently, it is postulated that the virus determines a state of hyper-inflammation in pulmonary tissue variously designated as macrophage activation syndrome (MAS), cytokine storm, and secondary hemophagocytic lymphohistiocytosis (sHLH) (Barth et al., 2020a; McGonagle et al., 2020a; Ruan et al., 2020). In particular, the cytokine storm induced by the activation of a large number of white cells (i.e. B cells, T cells, NK cells, macrophages) and resident tissue cells (i.e. epithelial and endothelial cells) is a common feature of severe COVID-19 cases, as it is responsible for ARDS and multiorgan failure (Azkur et al., 2020).

Notably, the post-mortem evidence regarding both the lung tissue damage supporting ARDS and the pulmonary vascular thrombotic findings might be consistent with the conclusion of a recent clinical prospective observational study. Such study reports that patients with a combination of very high D-dimer concentration and low lung static compliance due to COVID-19 have a dramatic increase in mortality rate (Grasselli et al., 2020). Thus, a critical prognosis could be considered for patients with pulmonary cells and vascular system affected by SARS-CoV-2.

Moreover, the data emerging from the literature review lead to consideration of the SARS-CoV-2 infection no longer as a disease almost exclusively affecting the lungs, but as a disease that affects multiple organ systems.

The heart findings in subjects who have died from COVID-19 are acute inflammatory signs such as focal lymphocytic infiltrates, individual cardiomyocyte injury, acute myocardial infarction, lymphocytic myocarditis/epicarditis/pericarditis (Hanley et al., 2020b; Buja et al., 2020; Lax et al., 2020; Menter et al., 2020; Rapkiewicz et al., 2020; Remmelink et al., 2020; Schaller et al., 2020). These tissue alterations could cause both cardiomyopathy and heart failure characterizing the potential natural evolution of COVID-19 as suggested by some authors (Yancy and Fonarow, 2020). Previous studies revealed that the dysregulated inflammatory response related to the failure of infection control process can affect the heart determining septic cardiomyopathy characterized by myocardial depression (Ventura Spagnolo et al., 2018). Coronary artery atheroma and/or atherosclerosis were other pathological findings that might cause an increased susceptibility to the negative consequences of the SARS-Cov-2 infection, including death (Ventura Spagnolo et al., 2018). Additionally, the endothelial small vessels damage and thrombosis were also observed in this organ, even if these were found in just a few cases (Hanley et al., 2020b; Nunes Duarte-Neto et al., 2020; Oprinca and Muja, 2020; Rapkiewicz et al., 2020). However, more interesting data emerged from myocardial ultrastructural analysis, revealing the presence of a small group of viral particles or single particles within the damaged interstitial cells of the myocardium, demonstrating loss of plasmalemma integrity (Tavazzi et al., 2020). ISH also showed the presence of the virus in interstitial cells and macrophage infiltrates (Lindner et al., 2020). This evidence supports the thesis that the direct viral infection of the heart could be another possible cause of myocardial damage (Bonow et al., 2020).

The microscopic liver analysis revealed in some cases lymphocytic infiltrates, especially in the portal/periportal tract and focal lobular inflammation with infiltration of predominant lymphocytes and neutrophils along with Kupffer cell hyperplasia and hypertrophy (Wang et al., 2020a; Lax et al., 2020; Nunes Duarte-Neto et al., 2020; Oprinca and Muja, 2020; Prilutskiy et al., 2020; Schaller et al., 2020; Varga et al., 2020). The described hepatocyte necrosis can be more likely attributed to hypoxia and systemic inflammation in severe cases (Horvatits et al., 2019). Microthrombi were observed by Rapkiewicz et al. (2020) in hepatic sinusoids. Interesting vascular findings were also reported by Sonzogni et al. (2020), such as portal vein endotheliitis and diffuse alterations of intrahepatic vascular structures together with luminal thrombosis. Also, besides the heart, the TEM analysis revealed the presence of typical coronavirus particles in the cytoplasm of hepatocytes with most viral particles without membrane-bound vesicles (Wang et al., 2020a).

The most frequently described alteration in kidney tissue was acute tubular damage (Hanley et al., 2020b; Kudose et al., 2020; Lax et al., 2020; Menter et al., 2020; Nunes Duarte-Neto et al., 2020; Oprinca and Muja, 2020; Santoriello et al., 2020; Sekulic et al., 2020; Su et al., 2020), which was described as flattened tubular epithelium and lumens containing sloughed epithelial lining cells, granular casts, Tamm-Horsfall protein, and intraluminal accumulation of cellular debris in focal areas (Yan et al., 2020). This type of alteration seems to be related to hypoxia and responsible for terminal renal failure, rather than directly associated with the virus, although the tubular epithelium has a high expression of ACE-2 receptors (Harmer et al., 2002; Ding et al., 2004). However, renal SARS-CoV-2 replication could potentially contribute to acute kidney injury (Menter et al., 2020). Despite this, the presence of SARS-CoV-2 in kidney tissue was observed by both TEM and immunofluorescence staining (Bradley et al., 2020; Rapkiewicz et al., 2020; Su et al., 2020). Moreover, microthrombi were observed in kidney vessels, especially in glomeruli (Hanley et al., 2020b; Nunes Duarte-Neto et al., 2020; Oprinca and Muja, 2020; Rapkiewicz et al., 2020; Santoriello et al., 2020; Su et al., 2020).

Not many research groups studied the brain in COVID-19 deceased patients. In some cases, researchers found that the encephalic small vessels were affected, and they described inflammatory lymphocytic infiltrates and hemorrhagic foci in the perivascular setting (Nunes Duarte-Neto et al., 2020; Hanley et al., 2020b; von Weyhern et al., 2020; Reichard et al., 2020; Conklin et al., 2020). Although they had some histological aspects in common, none of the authors found specific elements to establish a direct correlation with the viral infection. Nevertheless, von Weyhern et al. (2020) documented lymphocytic pan-encephalitis and meningitis with diffuse petechial hemorrhage in the entire brain even if they did not observe significant endotheliitis on the TEM and reported that the hypoxic morphological changes were localized in brain areas most commonly susceptible to developing damage (i.e., neocortex, cerebellum, and brainstem). Similarly, the hypoxic changes observed by Solomon et al. (2020) have no specific characteristics correlated to the SARS-Cov-2 infection. Finally, Reichard et al. (2020) excluded an association between the larger hemorrhagic lesions and both the perivascular inflammation and small vessels necrosis, suggesting that these changes occurred in close chronological proximity to the patient’s death, hypothesizing micro-thromboembolic pathogenesis, possibly related to COVID-19, based on the observation of microscopic neocortical infarcts.

Interesting evidence was observed in the spleen: red pulp hemorrhage (Nunes Duarte-Neto et al., 2020; Prilutskiy et al., 2020; Rapkiewicz et al., 2020), splenitis (Menter et al., 2020; Nunes Duarte-Neto et al., 2020) and, particularly, white pulp depletion associated with lymphocyte depletion and lymphohistiocytosis (Lax et al., 2020; Nunes Duarte-Neto et al., 2020; Oprinca and Muja, 2020; Prilutskiy et al., 2020; Rapkiewicz et al., 2020). The decrease of white cells could be correlated to both the viral attack on immunocytes and the lymphopenia described as an important clinical finding and could be considered a criterion of infection severity (Wang F. et al., 2020; Qin et al., 2020). On the other hand, the lymphohistiocytosis supports the suggested role of HLH as an important factor involved in the progression of severe COVID-19 (McGonagle et al., 2020b; Mehta et al., 2020). Other spleen findings regarded the vessels, showing follicular arterioles endothelial changes, vasculitis, and arterial thrombus (Nunes Duarte-Neto et al., 2020). HLH and lymphocyte depletion was also observed in lymph nodes (Lax et al., 2020; Prilutskiy et al., 2020). The bone marrow findings highlighted reactive myelopoiesis, prominent hyperplasia of cytotoxic CD8-positive T cells, and histiocytic cells CD163+ (Menter et al., 2020; Prilutskiy et al., 2020). Interestingly, some researchers suggested that the spleen and lymph nodes lymphocytic depletion could be partially attributable to an endogenous increase of cortisol secretion by the fasciculated zone of the adrenal gland appearing hyperplastic (Lax et al., 2020), as previously reported for lymphopenia related to SARS-1 disease (Chong et al., 2004; Panesar, 2008). Furthermore, the role of adrenal gland in sepsis is well established as part of an intrinsic neuroendocrine response to sepsis-related circulatory failure (Cumming et al., 1988; Ventura Spagnolo et al., 2019a; Ventura Spagnolo et al., 2019c). On the contrary, other researchers observed an evident adrenal vascular damage, namely vascular necrosis and endothelial apoptosis, hypothesizing that adrenal insufficiency related to vasculopathy may contribute to the cytokine storm characterizing COVID-19 (Iuga et al., 2020). Thus, this hypothesis could be supported by the patchy areas of infarct-type adrenocortical necrosis and organizing microthrombi in adrenal vessels described by Hanley et al. (2020b).

The digestive tract involvement, except for the liver, was little discussed, resulting in only mesenteric ischemia, small bowel sub-mucosal vessels endotheliitis, and pancreatitis (Hanley et al., 2020b; Lax et al., 2020; Remmelink et al., 2020; Varga et al., 2020). A research group made a very interesting consideration, evaluating only bowel ischemic damage and lung injuries and pointing out that the intestine lesions frequently do not correlate with tissue virus detection: RT-PCR positivity in cases without ischemia, and RT-PCR negativity in cases with ischemia (Skok et al., 2020). However, clinical reports described the recurrence of intestinal ischemia in COVID-19 patients with gastrointestinal symptoms noting an increased mortality rate with respect to patients with SARS-CoV-2–negativity (Norsa et al., 2020).

Two different studies (Nunes Duarte-Neto et al., 2020; Yang et al., 2020) described macroscopic and histological testis findings with results compatible with viral orchitis, characterized by lymphocyte inflammation with a prevalence of T-cells. Moreover, Yang et al. (2020) also observed the swelling of Sertoli cells and reduced Leydig cells, two cell populations with a strong expression of ACE-2 receptors.

As for the skin involvement, purpuric lesions were observed in association with other microscopic findings such as a microvascular endothelial and thrombotic damage and perivascular inflammatory infiltrate (Magro et al., 2020; Nunes Duarte-Neto et al., 2020). It must also be highlighted that the complement components analyzed by Magro et al. (2020) in lung tissue were detected in skin samples as extensive endothelial and subendothelial deposits of C5b-9.

The molecular analysis performed by several researchers using RT-PCR on post-mortem samples showed valuable data leading to different considerations. This investigation is fundamental to assess the SARS-CoV-2 infection and, as suggested by other authors (Sessa F. et al., 2020), it should always be performed, especially in probable and suspected cases. The present literature review confirmed that the virus is located mainly in lungs and upper airway tissue (Aguiar et al., 2020; Konopka et al., 2020a; Wang C. et al., 2020; Bradley et al., 2020; Tian et al., 2020b; Buja et al., 2020; Cipolloni et al., 2020; Craver et al., 2020; Edler et al., 2020; Fitzek et al., 2020; Fox et al., 2020; Kantonen et al., 2020; Karami et al., 2020; Lacy et al., 2020; Martines et al., 2020; Menter et al., 2020; Nunes Duarte-Neto et al., 2020; Oprinca and Muja, 2020; Schaller et al., 2020; Sekulic et al., 2020; Wichmann et al., 2020; Youd and Moore, 2020). The negativity reported in some cases could be explained by: 1) the early “window” period in which RT-PCR cannot detect the RNA; 2) the different clinical and biological evolution of the infection in the late phase resulting in subjects both negative or continuously positive; 3) the immune interindividual variability and the different virus haplotype (Sessa F. et al., 2020; Carter et al., 2020; Kucirka et al., 2020; Lescure et al., 2020; Norsa et al., 2020; Shen et al., 2020; Yi, 2020). Other factors affecting the RT-PCR results can be the sampling technique and post-mortem tissue degradation (Ventura Spagnolo et al., 2019b; Stassi et al., 2020; Youd and Moore, 2020). Moreover, this investigation was performed on other organs, resulting in positivity in the heart, liver, spleen, kidney, and testis of few cases (Wichmann et al., 2020; Bradley et al., 2020; Sekulic et al., 2020; Menter et al., 2020; Hanley et al., 2020b; Tian et al., 2020b; Lindner et al., 2020; Iuga et al., 2020], supporting that the SARS-CoV-2 might determine a direct effect not only on respiratory tissue. Moreover, Remmelink et al. (2020) reported virus detection in the heart, spleen, liver, kidney, bowel, and brain even if the histological findings were non-specific. Similar considerations regarded the study of Skok et al. (2020), which described bowel histological damage in both cases with and without virus RNA tissue detection.

The summarized evidence collected from the articlesincluded in this review show that COVID-19 is strictly related to a hyperinflammatory state that seems to start with DAD and immuno-thrombotic microangiopathy. Massive activation of the immune system and microvascular damage might also be responsible for indirect damage caused to other organs, even if the direct effect of the virus on these tissues cannot be excluded.

Research is still at an early stage in understandinghow SARS-CoV-2 both affects the immune system and determines organ damage, especially lung, but a better understandingisextremely importantin order to develop better(s)elective treatment strategies (Sessa L. et al., 2020). To date, in fact, there is yet no specific and standardized treatment even if *in vitro* studies and/or clinical trial of some antiviral drugs (such as inhibitors of RNA-dependent RNA polymerase, viral entry inhibitors, and viral fusion inhibitors) showed encouraging results (Kabir et al., 2020). Moreover, in this context, studies aimed at evaluating the genetic diversity of SARS-CoV-2 appear to be crucial for the potential association with virus conformational structural changes and, thus, an altered antigenicity supporting different immune responses (Kabir et al., 2020).

The strong points of the present review of literature are the autopsy reports, 1) providing a fundamental base in the process of understanding the infection consequences, 2) demonstrating that the post-mortem investigations (gross examination, histological and immunohistochemical analysis, electron microscopy, and molecular test) are non-replaceable tools for various reasons. This way, it is possible to assess if the subject died “from” or “with” COVID-19, ensuring reliable epidemiological, pathological, molecular, and global health data. Our study has limitations, mainly related to the unknown validity of pathological assessment of macroscopic and microscopic findings; actually, there is no published risk of bias assessment tool that could be applied to the material and methods analyzed in this review.

Another limitation was missing details regarding the prevalence of the detected features stratified through the characteristics of the subjects, such as age, gender, race, and comorbidities and their pharmacological treatment. Additionally, only data available on PubMed database were taken into account. Finally, limitations secondary to paucity of performed autopsies and data available in literature did not allow a reliable estimation to be conducted of the overall quality of the included studies through the statistical evaluation of the pathological findings rate.

All post-mortem findings, along with clinical pictures, are useful to better understand pathogenesis and pathophysiology, and, consequently, the best therapeutic management of patients. Therefore, this is the right time “to reverse the decline of autopsy rates”, as affirmed by De Cock et al. (2019) a few months before the COVID-19 outbreak, in order to benefit global public health. As observed by Barth et al. (2020b), a “call to action” for more autopsy reports containing detailed findings is necessary.

## Data Availability

The original contributions presented in the study are included in the article/Supplementary Material, further inquiries can be directed to the corresponding author.
